# Physiological random processes in precision cancer therapy

**DOI:** 10.1371/journal.pone.0199823

**Published:** 2018-06-29

**Authors:** Nick Henscheid, Eric Clarkson, Kyle J. Myers, Harrison H. Barrett

**Affiliations:** 1 Center for Gamma-Ray Imaging, University of Arizona, Tucson, AZ, United States of America; 2 Program in Applied Mathematics, University of Arizona, Tucson, AZ, United States of America; 3 Department of Medical Imaging, University of Arizona, Tucson, AZ, United States of America; 4 College of Optical Sciences, University of Arizona, Tucson, AZ, United States of America; 5 Center for Devices and Radiological Health, Food and Drug Administration, Silver Spring, MD, United States of America; Universita degli Studi di Catania, ITALY

## Abstract

Many different physiological processes affect the growth of malignant lesions and their response to therapy. Each of these processes is spatially and genetically heterogeneous; dynamically evolving in time; controlled by many other physiological processes, and intrinsically random and unpredictable. The objective of this paper is to show that all of these properties of cancer physiology can be treated in a unified, mathematically rigorous way via the theory of random processes. We treat each physiological process as a random function of position and time within a tumor, defining the joint statistics of such functions via the infinite-dimensional characteristic functional. The theory is illustrated by analyzing several models of drug delivery and response of a tumor to therapy. To apply the methodology to precision cancer therapy, we use maximum-likelihood estimation with Emission Computed Tomography (ECT) data to estimate unknown patient-specific physiological parameters, ultimately demonstrating how to predict the probability of tumor control for an individual patient undergoing a proposed therapeutic regimen.

## Introduction

In a seminal paper in 2000, Hanahan and Weinberg [1] identified six traits that are hallmarks of most if not all cancers. Malignant neoplasms must be able to proliferate without external growth factors; evade growth suppressors; resist cell death; replicate indefinitely; produce new blood vessels, and activate invasion and metastasis. In a later paper [2] these same authors added two other hallmarks—reprogramming energy metabolism and evading destruction by the immune system—and they noted the crucial role played by the tumor microenvironment.

Each of these traits is associated with a complex set of genetically regulated physiological processes [3]. For example, the state of oxygenation within a tumor is a crucial factor in the development and treatment of cancer. In tumors, the protein HIF-1-*α* (hypoxia-inducible-factor 1-*α*) regulates metabolism and promotes angiogenesis in response to hypoxia. Hypoxia itself, on the other hand, leads to invasion and resistance to radiation or chemotherapy. The oxygen content of a tumor can be quantified by the partial pressure, pO_2_, or the oxygen saturation SaO_2_ (ratio of oxygenated to total hemoglobin).

In chemotherapy, the spatial and temporal distribution of the drug and the response of the tumor to the drug are critical physiological processes. The response can be defined by the change in mitosis (cell division) and apoptosis (programmed cell death) after therapy. In normal tissues, these two processes are closely balanced, but cancer is characterized by an increased rate of mitosis and/or a reduced rate of apoptosis. Conversely, anticancer drugs can either inhibit mitosis or stimulate apoptosis.

It is generally agreed in the literature that the spatial and genetic heterogeneity of these physiological processes plays a crucial role in initiating the Hanahan-Weinberg traits and determining their magnitudes [4–8], but there has been less effort towards defining physiological heterogeneity in rigorous mathematical terms or describing the interactions between different heterogeneous processes. The first goal of this paper is to introduce a framework for filling this gap.

**Fig 1:**
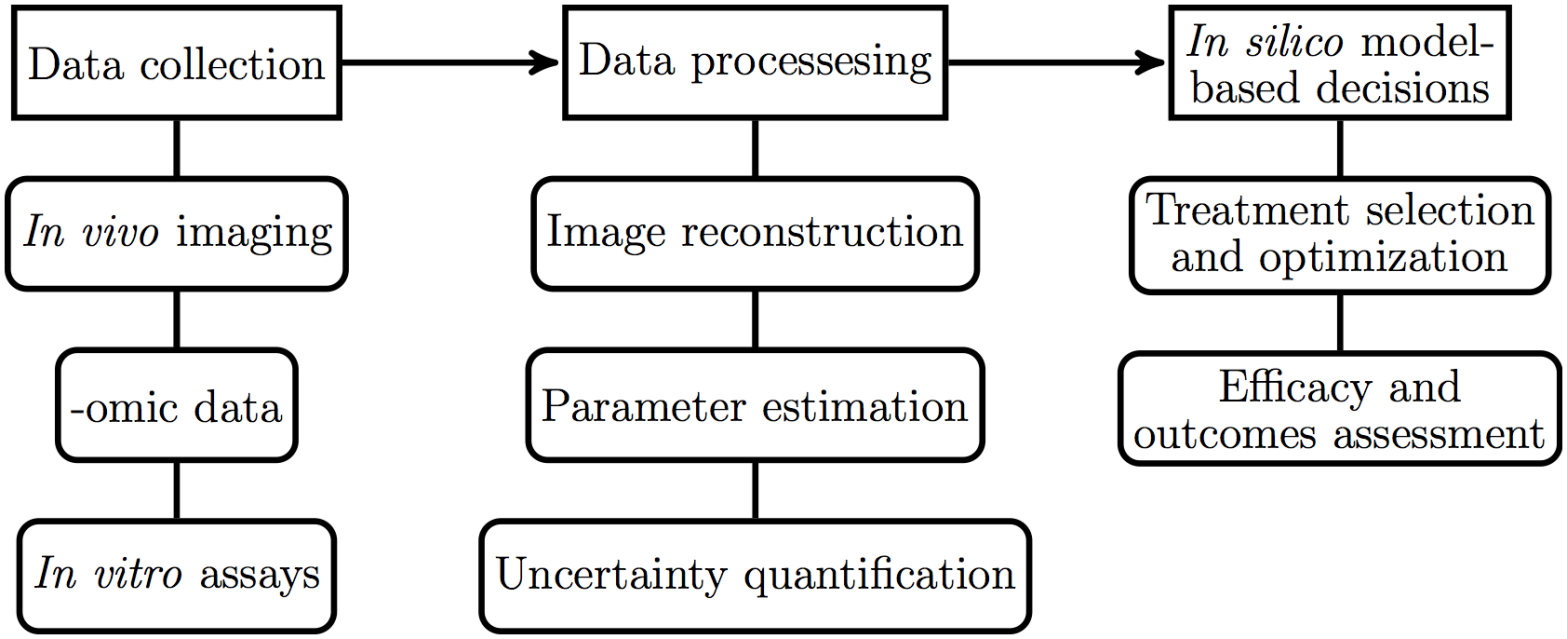
Information flow in precision medicine. The information pipeline for precision medicine involves collecting patient-specific data, using this data to personalize validated mathematical and computational models, then using the output of such models to perform treatment selection, optimization, and assess efficacy.

Without detailed information about the specific nature and degree of heterogeneity for a given patient, prediction of clinical outcomes is difficult. We thus also provide a theoretical framework for making patient-specific measurements of heterogeneous physiological processes via molecular imaging. As measurement devices are inherently noisy and their data incomplete, estimation of patient-specific processes and parameters must be accompanied by quantification of uncertainty. We thus also aim to provide a statistically rigorous way to quantify uncertainty in estimates of patient-specific, heterogeneous physiological processes and hence any clinically relevant decision parameters derived from such estimates. In this sense, we are providing a statistical framework for precision medicine in the context of spatiotemporal physiological quantities and noisy data, where we define precision medicine as the general tactic of combining patient-specific data, mathematical and statistical modeling and computational techniques to assist in the clinical decision-making process; refer to Fig 1.

In light of these issues, we adopt the viewpoint that physiological processes can be described rigorously as spatiotemporal *random processes*, in a mathematical sense. To be more precise, one realization of a physiological random process (say for one cancer patient) is a function of position in the body and time. We can specify the spatial position by Cartesian coordinates (*x*, *y*, *z*) or, equivalently, by a three-dimensional (3D) vector ***r*** with these components. A general function of space and time is denoted as *f*(***r***, *t*), and if it is a random, unpredictable function, then it is one realization of a random process, which is to say it is an element of an *ensemble* of functions, denoted as {*f*(***r***, *t*)}. In this paper the processes are physiological, and *f*(***r***, *t*) typically specifies the magnitude of the physiological effect (e.g., pO_2_ in our oxygenation example or apoptosis and mitosis rates) at location ***r*** and time *t*. There are two types of ensembles that are relevant to cancer physiology. First, as each patient can be considered as a sample drawn from a population, we can consider a population-level ensemble. For example, the ensemble {*f*(***r***, *t*)} for the oxygenation example would consist of a large set of possible functions *f*(***r***, *t*) describing the spatiotemporal concentration of oxygen in a well-defined region of the body, and a realization of this process *f*_*j*_(***r***, *t*) corresponds to a patient (*j*) drawn at random from the population. Second, due to uncertainties inherent in the data collection and processing steps, each individual patient gives rise to an ensemble of functions which are consistent with the data collected. These ensembles could be called *prior* and *posterior* ensembles, though we do not necessarily imply the usage of Bayesian techniques [9].

We distinguish random functions from random variables or random vectors. A random variable, such as an image feature, is a single random scalar, and a finite set of random variables, such as a digital image or a set of image features, is a finite-dimensional random vector. For any random process {*f*(***r***, *t*)}, one may derive a host of finite-dimensional statistical quantities of interest. For example, observing the average value of *f*(***r***, *t*) over a spatial region of interest *V* and time interval [*t*_0_, *t*_1_] gives rise to a scalar random variable.

**Fig 2:**
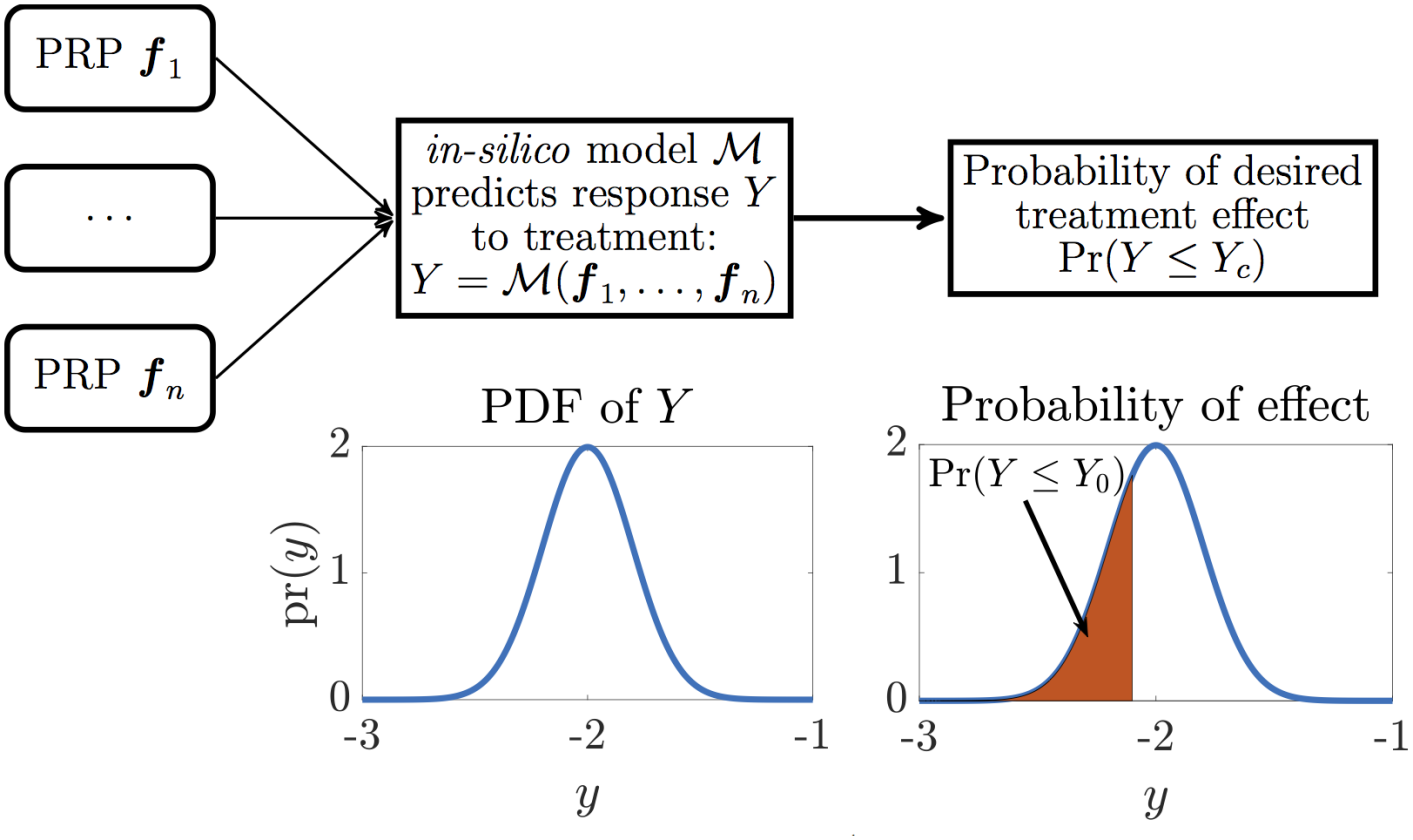
Modeling uncertainty *in-silico*. The paradigm of using *in-silico* modeling to make predictions about treatment effect under uncertainty about the physiological processes involved. Relevant physiological processes ***f***_1_, …, ***f***_*n*_ are identified, and a model M predicting response is selected. Because of uncertainty in the processes, they are modeled as random; hence the response *Y* is random, having PDF pr(*y*). A desired effect is identified, for instance response less than a threshold *Y*_*c*_. Its probability is the area under pr(*y*) left of *Y*_*c*_.

In this paper, we will derive a particular scalar random variable *Y* related to cancer treatment efficacy. The variable *Y* is expressed as a function of several physiological processes related to drug delivery and effect. By modeling the input processes as random, the variable *Y* is made random, permitting the definition of a *probability* of some desired effect, in this case spatially averaged, short-term tumor control; refer to Fig 2 for a block-level illustration. We will demonstrate how the probability distribution of *Y* is related to the statistics of the input random processes, then show how imaging data can be used to construct a patient-specific probability distribution for *Y*. This will lead to an explicit expression for a patient-specific probability of tumor control which can be computed using clinically available data and the statistical techniques outlined in this paper.

### Characteristic functionals

While the statistical properties of random variables and random vectors are easily specified by probability density functions (PDFs), describing the statistical properties of a random process requires the application of more sophisticated tools. A random process consists of a set of functions {*f*(***r***, *t*)}, each of which can be regarded as an element of an infinite-dimensional vector space, so it is not usually convenient to define conventional PDFs for them (see S1 Appendix for a discussion of this issue). Nevertheless, all the statistical properties of a random process can be derived from its *characteristic functional*, a powerful mathematical tool which to our knowledge has not previously been applied to oncology or precision medicine. A functional accepts a function as its input and returns a single scalar value, and we define the characteristic functional for a physiological random process consisting of functions {*f*(***r***, *t*)} by

$$\begin{array}{c} \Psi_{f}\;\left[\phi \left(r\right),t\right]\equiv \left\langle \exp\left(-2\pi i\int_{V}d^{3}r\;\phi \left(r\right)f\left(r,t\right)\right)\right\rangle \;, \end{array}$$ (1)

where *ϕ*(***r***) is an arbitrary real-valued test function and the integral is over the volume *V* of a tumor or some other region of interest. The angle brackets denote an average over all possible realizations of the random process for some ensemble of patients or subjects. The functional (1) contains a complete description of the spatial statistics of the process at any given time; the time variable *t* in Eq (1) allows these spatial statistics to evolve as the disease progresses or therapeutic interventions take effect, but it is not intended to capture joint statistical variations across multiple times, so the test function *ϕ*(***r***) is not a function of time. An analogous characteristic functional can be defined when temporal correlations are of interest; see S1 Appendix.

For a survey of the mathematical properties of random processes and characteristic functionals, see [10, 11] and S1 Appendix. One of the key advantages of the characteristic functional is that there are many general classes of random processes for which Ψ_***f***_ is known analytically, which makes it possible to compute any of the conventional statistical properties, such as moments or marginal probability density functions. This includes many classes of non-Gaussian processes which are typically challenging to manipulate—see [10, 11], and also S2 Appendix. Moreover, the characteristic functional can be applied to personalized medicine by collecting molecular images or other data from a particular patient and using them to estimate any parameters that appear in a patient-specific characteristic functional [12].

### Emission computed tomography

A general method for measuring physiological random processes is Emission Computed Tomography (ECT), defined broadly as three-dimensional (3D) imaging of molecules or cells that have been labeled so that they emit light, high-energy photons or charged particles without significant alteration of their biological function. The labeling can use radionuclides or light-emitting molecules, so the emissions can be nuclear decay products, including electrons, positrons and high-energy photons, or visible or near-infrared photons.

The most familiar forms of ECT are SPECT (Single-Photon Emission Computed Tomography) and PET (Positron Emission Tomography), in which molecules labeled with radionuclides are imaged. SPECT and PET are widely used in clinical medicine and in preclinical studies with small animals. There is considerable interest in pushing the spatial resolution of radionuclide imaging ever finer, especially in SPECT where intrinsic detector resolutions around 50 *μ*m have been reported from several labs, and reconstructed resolutions for small-animal SPECT are approaching 100 *μ*m [13].

Much finer resolution in radionuclide imaging can be obtained with autoradiography, which uses the charged particles emitted by a radionuclide (alpha particles, beta particles or positrons) rather than the high-energy photons. Classical autoradiography is carried out by placing a thin slab of excised tissue on film or an electronic detector and taking a long exposure. More modern approaches use particle detectors operating at video rates [14]. Images with spatial resolution around 5 *μ*m and sub-nanoCurie sensitivity can be obtained ex vivo with excised slices, and 3D images can be synthesized by stacking the slice images in a computer.

In-vivo charged-particle autoradiography can be performed at high resolution with endoscopic devices, in mouse window chambers or for superficial lesions clinically, but to date only without tomography. Recent work has shown the possibility of true ECT with charged particles in vivo [15]. We refer to the general method as CPET (Charged-Particle Emission Tomography), with the special cases of BET (Beta-particle Emission Tomography) and *α*ET (Alpha-particle Emission Tomography).

Finally, there is great interest in OpET (Optical Emission Tomography), where the radionuclide label is replaced by a fluorescent or bioluminescent molecule. Fluorescence imaging of cells in culture has been the mainstay of molecular biology, and immunohistochemistry (IHC) with fluorescent antibodies is the foundation of modern pathology.

There are numerous ways to produce 3D images with optical emitters. At the clinical scale, fluorescence-enhanced optical imaging of the breast or lymphatic vessels [16, 17] yields 3D reconstructed images with a resolution around a millimeter. Several commercial preclinical imaging systems collect light emerging at different angles from a mouse or other small animal and reconstruct OpET images. There is also a burgeoning interest in various forms of 3D fluorescence imaging at the microscopic scale; these include deconvolution methods, scanning confocal microscopes, optical projection tomography [18] and 3D superresolution methods derived from the Nobel-prize-winning work of Betzig, Hell and Moerner [19, 20].

For all of these ECT modalities and for almost any chosen physiological random process, many different labeling agents (tracers) have been developed [21, 22].

A physiological random process ***f*** = *f*(***r***, *t*) imaged via molecular imaging will give rise to a random data set **g**, usually taken to be a finite-dimensional random vector. In the population ensemble picture, we view the pair {***f***, **g**} as statistical covariates. As patient *j* gives rise to a realization *f*_*j*_(***r***, *t*), the conditional random vector **g**|***f***_*j*_ represents the random data vector for patient *j*. The statistics of **g**|***f***_*j*_ are related to the physics of imaging, which we will not discuss here; see e.g. [10]. A major goal of this paper is to show how ECT data and characteristic functionals can be used to study the physiological random processes that influence outcomes in cancer chemotherapy.

We first discuss how classical models of tumor growth, drug delivery and response to therapy can be modified into spatiotemporal models involving Physiological Random Processes (PRPs). The goal of this exercise is to demonstrate that under the PRP hypothesis, it is possible to derive clinically relevant parameters that are related to the prediction of treatment response for individual patients. Later, we discuss how ECT data can be used to perform statistical estimation of unknown parameters in these models and demonstrate how the theory of maximum likelihood estimation allows for the quantification of uncertainties in these estimates.

## Tumor growth and response to therapy

### Classical models

There is an extensive literature on mathematical models for tumor growth; common mathematical forms are exponential, logistic, Gompertz and von Bertalanffy [23–30]. There is another extensive literature on the response of cancer cells to chemotherapy drugs, radiation or other interventions [31–37]. For simplicity we will consider only avascular and premetastatic growth; incorporating both angiogenesis and metastasis requires more complex mathematical models.

Many of the papers cited present ordinary differential equations for the time dependence of the total number of clonogenic cells in a tumor, denoted *N*(*t*). It is common to regard *N*(*t*) as a deterministic quantity that can be found by solving the differential equations, with no spatial inhomogeneity, uncertainty or stochastic effects considered. The differential equations always involve a small number of free parameters, which can be fixed by fitting the predicted behavior of *N*(*t*) to measurements on tumors in humans or animals.

These investigations often result in equations having the generic form

$$\begin{array}{c} \frac{dN(t)}{dt}={\frac{dN(t)}{dt}|}_{\text{growth}}+{\frac{dN(t)}{dt}|}_{\text{drug}}\;, \end{array}$$ (2)

where the first term describes the growth of the tumor in the absence of a therapeutic intervention and the second accounts for a chemotherapy drug.

**Fig 3:**
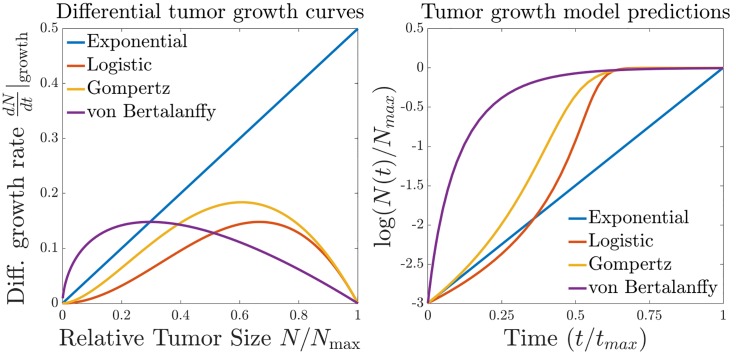
Tumor growth curves. A comparison of several differential growth curves of the form *N*Φ(*N*). Note that in each case except unbounded exponential growth, the differential growth rate goes to zero as the tumor approaches its maximum size.

The simplest form for the growth term is *dN*(*t*)/*dt*|_growth_ = *βN*(*t*) (where *β* is a positive constant), which leads to exponential growth, *N*(*t*) ∝ exp(*βt*). It is widely observed, however, that the growth rates of solid tumors decrease as the tumors get larger, and hence several common models take the form *dN*(*t*)/*dt*|_growth_ = *N*(*t*)Φ[*N*(*t*)], where Φ(⋅) is a monotonically decreasing function. The resulting differential growth rates for several common models are shown in Fig 3. For example, the commonly used Gompertzian model assumes that the tumor size reaches a “carrying capacity” *N*_max_ limited by blood supply and nutrients. The Gompertz model enforces this condition by taking Φ[*N*(*t*)] ∝ −ln(*N*(*t*)/*N*_max_). Mechanistic explanations for Gompertzian tumor growth have been given by Gyllenberg [38, 39], Hahnfeldt [40] and Norton [24].

A common form for the drug effect in (2), derived from the classical law of mass action, assumes that *dN*(*t*)/*dt*|_drug_ = −*αC*(*t*)*N*(*t*), where *C*(*t*) is the drug concentration and *α* is positive. Equivalently,

$$\begin{array}{c} \frac{1}{N(t)}{\frac{dN(t)}{dt}|}_{\text{drug}}=\frac{d}{dt}\ln N\left(t\right)=-\alpha C\left(t\right)\;. \end{array}$$ (3)

This form, often called *linear log-kill*, says that the fractional rate of cell killing is a linear function of the drug concentration and is usually attributed to Skipper [41]. Linear log-kill is a reasonable model for certain cell-cycle-independent drugs such as doxorubicin, at least *in vitro* [42]. More generally, cytotoxic drugs can affect cell populations nonlinearly with respect to *C*(*t*), for instance due to saturation effects and cell cycle inhomogeneities. We discuss several nonlinear response models later; refer to Eqs (32) and (37).

With the general growth model *dN*(*t*)/*dt*|_growth_ = *N*(*t*)Φ[*N*(*t*)] and the mass-action assumption for the drug effect, the overall model (2) becomes

$$\begin{array}{c} \frac{d}{dt}\ln N\left(t\right)=\Phi \left[N\left(t\right)\right]-\alpha C\left(t\right)\;. \end{array}$$ (4)

With the choice of Gompertzian growth kinetics,

$$\begin{array}{c} \frac{d}{dt}\ln N\left(t\right)=-\mu \ln\left(\frac{N(t)}{N_{\text{max}}}\right)-\alpha C\left(t\right)\;. \end{array}$$ (5)

Eq (5), paired with an initial condition of *N*(0) = *N*_0_ ≥ 0 total cells, describes the deterministic evolution of *N*(*t*). Again, neither spatial effects nor uncertainty are addressed in such a model. We will address both by replacing *N*(*t*) with a corresponding physiological random process *n*(***r***, *t*).

### Tumor growth and response as random processes

We can define a continuum density of cells, denoted *n*(***r***, *t*), which has units of number of cells per unit volume. In the viewpoint of this paper, *n*(***r***, *t*) is a spatial random process that evolves in time, and *N*(*t*) is now viewed as a time-dependent random variable. The two random entities are related by

$$\begin{array}{c} N\left(t\right)=\int_{V(t)}d^{3}r\;n\left(r,t\right)\;, \end{array}$$ (6)

where *V*(*t*) is the evolving tumor volume. If we are interested in the means of these random quantities (statistical expectations with respect to some ensemble of tumors), we can indicate the means by overbars and write

$$\begin{array}{c} \bar{N}\left(t\right)=\int_{V(t)}d^{3}r\;\bar{n}\left(r,t\right)\;. \end{array}$$ (7)

This result depends only on the linearity of statistical expectations, not on any particular statistical properties of *n*(***r***, *t*) or *N*(*t*).

We should not necessarily expect $\bar{n}\left(r,t\right)$ to be spatially uniform to any reasonable approximation: malignant cells may be fairly widely separated in a tumor, spaced by the extracellular matrix and interspersed with fibroblasts, lymphocytes and other cells in the tumor microenvironment. Moreover, tumor cells often tend to grow in clusters called nests or islets, surrounded by a collagenous shell.

We postulate that a reasonable random-process counterpart of Eq (4) is

$$\begin{array}{c} \frac{\partial }{\partial t}\ln n\left(r,t\right)=\Phi \left[n\left(r,t\right)\right]-\alpha \left(r\right)c\left(r,t\right)\;, \end{array}$$ (8)

where now *n*(***r***, *t*), *α*(***r***, *t*) and *c*(***r***, *t*) are treated as random processes. The growth nonlinearity Φ may also depend on some auxiliary random process parameters; for example, with a Gompertzian model, we would have

$$\begin{array}{c} \frac{\partial }{\partial t}\ln n\left(r,t\right)=-\mu \left(r\right)\ln\left(\frac{n(r,t)}{n_{\text{max}}\left(r\right)}\right)-\alpha \left(r\right)c\left(r,t\right)\;. \end{array}$$ (9)

where the growth rate *μ*(***r***), carrying capacity *n*_max_(***r***) and drug sensitivity *α*(***r***) are all allowed to be spatial random processes. We assume further that the initial condition *n*(***r***, 0) is strictly positive at all points within the potential tumor volume: in mechanistic terms, we assume tumor growth proceeds from pre-existing clonogenic cells rather than spontaneous mutations, so it cannot begin in a region where there is zero density of malignant cells at *t* = 0.

Similarly, to be consistent with the idea of linear log-kill, we assume for the remainder of this section that the drug sensitivity *α*(***r***) is independent of time and statistically independent of the drug distribution *c*(***r***, *t*). More general response models can also be considered [43].

Another class of spatial growth models, which we will not discuss here, allows for diffusive and chemotactic migration of tumor cells; see e.g. [44–47].

**Fig 4:**
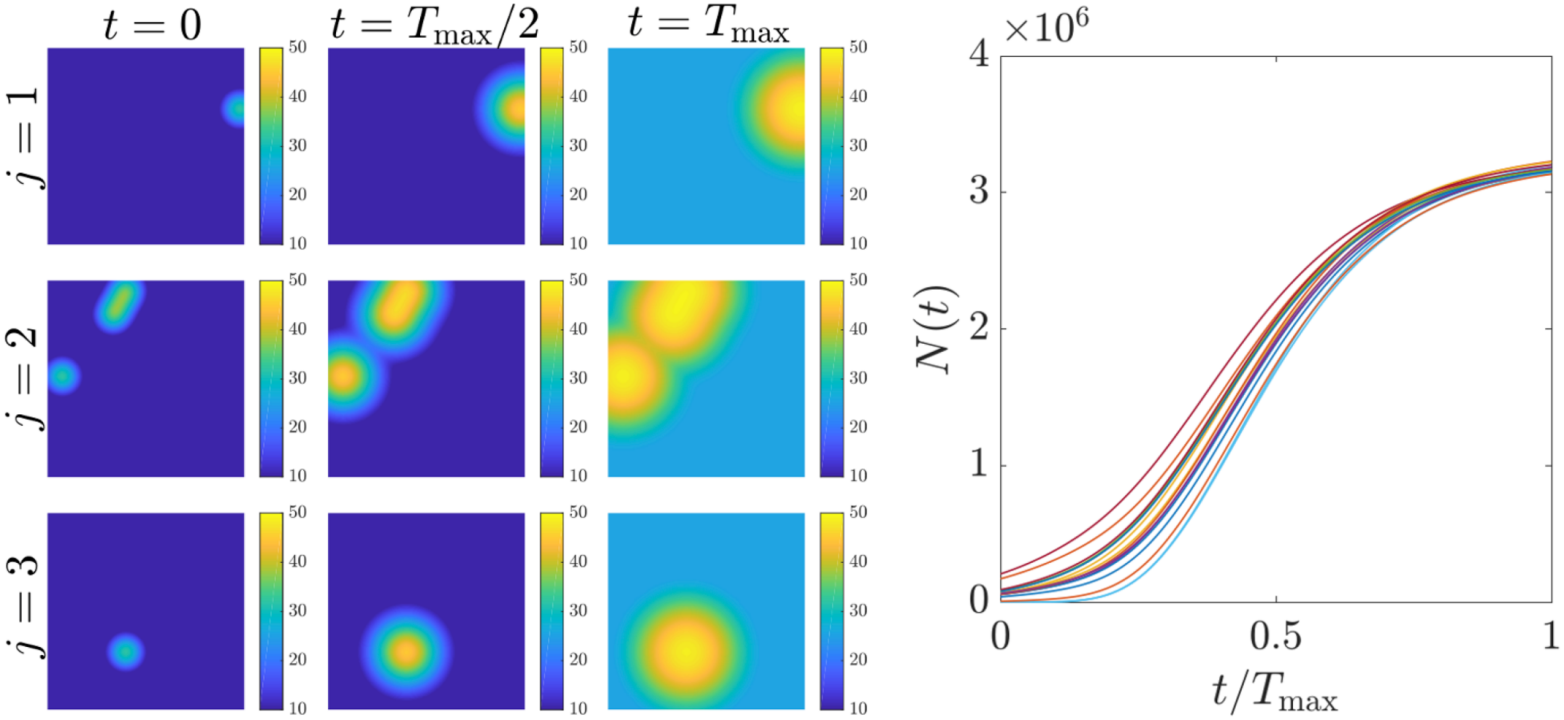
Tumor growth under uncertainty. A simulation of Gompertzian growth of the spatial random field *n*(***r***, *t*), as modeled in Eq (9) with *c*(***r***, *t*)≡0, is displayed on the left. In this simulation, both the local growth constant *μ*(***r***) and the local carrying capacity *n*_max_(***r***) are spatially constant but random; the initial condition *n*(***r***, 0) is taken as a so-called *lumpy background* random process (see S2 Appendix). Three realizations (rows) are shown at three times. The total cell number *N*(*t*) as defined in (6) is shown on the right for 16 realizations of the process.

Because each function in (9) is treated as a random quantity, we must specify the sense in which the equality holds. We will assume throughout that a differential equation such as (9) holds in the classical (or weak, in the sense of generalized functions) sense for each fixed realization of the random parameters. This assumption allows us to solve (9) using standard differential equation techniques, then derive statistical distributions for the resulting solution and any quantities of interest related to it. Such an equation is frequently called a *random* differential equation, to distinguish from *stochastic* differential equations which require more sophisticated solution strategies such as the Itô formalism (see discussion in [48], section 4.7). See Fig 4 for an illustration.

In S3 Appendix, we derive an expression for the evolution of the characteristic functional for ln *n*(***r***, *t*) under the hypothesis that the density of tumor cells is a lognormal random process (see S2 Appendix).

#### Tumor response

Chemotherapy drugs remain in a patient’s circulation for a few hours or days at most, and it is reasonable to assume that the tumor growth rate is negligible over this period. From (9) without the Gompertzian term, we can write

$$\begin{array}{c} \frac{\partial }{\partial t}n\left(r,t\right)=-\alpha \left(r\right)c\left(r,t\right)n\left(r,t\right)\;. \end{array}$$ (10)

This equation can also be related to the fundamental processes of cell division (mitosis) and cell death. Cell death can be by necrosis, autophagy or apoptosis (programmed cell death). There is also the process of cell-cycle arrest, which is mathematically equivalent to apoptosis.

If we consider only drug-induced apoptosis, we can write

$$\begin{array}{c} \frac{\partial }{\partial t}n\left(r,t\right)=u^{mit}\left(r,t\right)-u^{ap}\left(r,t\right)=-\alpha \left(r\right)c\left(r,t\right)n\left(r,t\right)\;, \end{array}$$ (11)

where the random processes *u*^*mit*^(***r***, *t*) and *u*^*ap*^(***r***, *t*) are rates per unit volume for mitosis and apoptosis, respectively. The difference of these two random processes can be interpreted as the overall proliferation rate per unit volume. In normal tissues the mitosis and apoptosis terms are in close balance (homeostasis), but in tumors there is a net growth in the absence of therapy. Chemotherapy drugs can decrease mitosis or increase apoptosis, either of which will cause a decrease in the overall proliferation rate ∂*n*(***r***, *t*)/∂*t*.

Integration of (10) or (11) over time yields a random process *y*(***r***), which can be interpreted as a pointwise log-kill:

$$\begin{array}{c} y\left(r\right)\equiv \ln\left(\frac{n(r,t_{0}+T)}{n(r,t_{0})}\right)=-\int_{t_{0}}^{t_{0}+T}dt\;\alpha \left(r\right)\;c\left(r,t\right)\;, \end{array}$$ (12)

where *T* is the time between drug administration and observation of the random process.

Now integrate both sides of (12) over a volume *V* that is large enough to encompass the tumor before and after the therapy. The result is a random variable *Y*, referred to as the *integrated log-kill* and given by

$$\begin{array}{c} Y\equiv \int_{V}d^{3}r\;\ln\left(\frac{n(r,t_{0}+T)}{n(r,t_{0})}\right)=-\int_{V}d^{3}r\;\alpha \left(r\right)\;\int_{t_{0}}^{t_{0}+T}dt\;c\left(r,t\right)\;. \end{array}$$ (13)

Because we are assuming for now that the drug sensitivity is independent of time, (13) becomes

$$\begin{array}{c} Y=-\int_{V}d^{3}r\;\alpha \left(r\right)\;AUC\left(r\right) \end{array}$$ (14)

where *AUC*(***r***) is the area under the curve of the drug concentration at point ***r*** vs. time:

$$\begin{array}{c} AUC\left(r\right)\equiv \int_{t_{0}}^{t_{0}+T}dt\;c\left(r,t\right)\;. \end{array}$$ (15)

Note that *AUC*(***r***) is still a spatial random process. If *T* is large enough for complete drug clearance from the tumor, *AUC*(***r***) is independent of *T* and can be interpreted as the total exposure of tumor tissue at point ***r*** to the drug.

The statistics of the random variable *Y* for a specified drug distribution ***c*** can be derived from the conditional characteristic *function* (not functional in this case because *Y* is a scalar), which is given in terms of the characteristic functional of ***α*** by

$$\begin{array}{ccc} \psi_{Y|c}\left(\xi \right)={\left\langle \exp\left(-2\pi i\xi Y\right)\right\rangle }_{Y|c} & = & {\left\langle \exp\left(2\pi i\xi \int_{V}d^{3}r\;\alpha \left(r\right)\;AUC\left(r\right)\right)\right\rangle }_{\alpha }\\ & = & \Psi_{\alpha }\left[-\xi \;AUC\left(r\right)\right]\;. \end{array}$$ (16)

We can then write the conditional probability density function for *Y* as

$$\begin{array}{c} \text{pr}\left(Y|c\right)=\int_{-\infty }^{\infty }d\xi \;\psi_{Y|c}\left(\xi \right)\exp\left(2\pi i\xi Y\right)=\int_{-\infty }^{\infty }d\xi \;\Psi_{\alpha }\left[-\xi \;AUC\left(r\right)\right]\exp\left(2\pi i\xi Y\right)\;, \end{array}$$ (17)

This is a fundamental equation in chemotherapy, relating the time-integrated drug distribution to the random logarithmic cell kill via the spatially varying statistics of the cellular sensitivity to the drug. Because pr(*Y*|***c***) is conditional on ***c***, (17) is directly applicable only when the drug distribution is known exactly, but its extension to the practical clinical case where the drug distribution is either unknown and random or estimated from noisy molecular imaging data is discussed later. The expression (17) also requires a characteristic functional Ψ_***α***_[***ϕ***] for the drug sensitivity, which can either be estimated from population statistics by fitting one of the models discussed in S2 Appendix to a population database [12], or derived from statistical analysis of patient-specific imaging data, as we discuss later.

#### Tumor control

One application of (17) is in specifying the efficacy of a drug treatment in terms of degree of short-term tumor control. If there is successful death of tumor cells immediately following therapy, *Y* is a negative number, so we *define* tumor control as achieving a *Y* that is less than some chosen threshold *Y*_*c*_ (e.g., *Y*_*c*_ = −3 for tumor reduction by a factor of *e*^3^ ≈ 20). The conditional probability of tumor control for a specified drug concentration is then given by

$$\begin{array}{ccc} & & \text{Pr}\left(TC|c\right)=\text{Pr}\left(Y<Y_{c}|c\right)=\int_{-\infty }^{Y_{c}}dY\;\text{pr}\left(Y|c\right)\\ & & \;=\int_{-\infty }^{Y_{c}}dY\int_{-\infty }^{\infty }d\xi \;\Psi_{\alpha }\left[-\xi \;AUC\left(r\right)\right]\;\exp\left(2\pi i\xi Y\right)\;, \end{array}$$ (18)

Both of these integrals are one-dimensional, so they are readily performed numerically if both *c*(***r***, *t*) and an analytic form for Ψ_***α***_[***ϕ***] are known.

To put this result into the context of chemotherapy, we assume that mass *M* of the drug has been administered to a patient. Within the confines of the linear model for the drug distribution used thus far, *c*(***r***, *t*) and hence *AUC*(***r***) are proportional to *M*, so we can define *AUC*(***r***) = *M* ⋅ *auc*(***r***), where *auc*(***r***) is *AUC*(***r***) for *M* = 1. Then we can write

$$\begin{array}{c} \text{Pr}\left(TC|c,M\right)=\int_{-\infty }^{Y_{c}}dY\int_{-\infty }^{\infty }d\xi \;\Psi_{\alpha }\left[-\xi M\cdot auc\left(r\right)\right]\;\exp\left(2\pi i\xi Y\right)\;. \end{array}$$ (19)

To extend any of the Eqs (17)–(19) formally to an ensemble of patients with different drug distributions, one can simply perform an average over ***c***; for example, (18) becomes

$$\begin{array}{c} \text{Pr}\left(TC\right)={\left\langle \text{Pr}\left(TC|c\right)\right\rangle }_{c}=\int_{-\infty }^{Y_{c}}dY\int_{-\infty }^{\infty }d\xi \;{\left\langle \Psi_{\alpha }\left[-\xi \;AUC\left(r\right)\right]\right\rangle }_{c}\exp\left(2\pi i\xi Y\right)\;. \end{array}$$ (20)

### Other response models

The formulas for tumor response developed above are all linear, in the sense that the log-kill is a linear function of the drug concentration, and local, in the sense that the response of the tumor at some point ***r*** depends on the drug concentration only at that point. There are situations in clinical oncology, however, where the response can be nonlinear, nonlocal or both. In this subsection we look at how these effects can be incorporated into a general theory of physiological random processes in oncology. The models presented can be used in combination with patient-specific imaging data to extract detailed spatiotemporal physiological information, which can subsequently be used to better predict treatment response.

#### Nonlocal processes with negligible time delay

As an example of nonlocal drug action, consider targeted radionuclide therapy, which uses radioactive drugs that bind to a specific receptor on tumor cells. While bound, the radioisotope in the drug undergoes radioactive decay, emitting gamma rays and charged particles. Depending on the isotope, the charged particles might include beta particles (high-energy electrons), positrons, conversion electrons or alpha particles. The energetic radiations kill cancer (and other) cells by damaging DNA, but for present purposes all we need to know is that the damage can be at some distance from the cell to which the drug molecule is bound; the range is tens of microns for alpha particles in tissue, millimeters for beta particles and positrons, and centimeters for gamma rays.

Because of the speed of the nuclear decay products, there is negligible time delay between the decay and the DNA damage. In that case the general linear but nonlocal model is obtained by the replacement

$$\begin{array}{c} \int_{V}d^{3}r\;\alpha \left(r,t\right)\;c\left(r,t\right)\to \int_{V}d^{3}r\;\alpha \left(r,t\right)\;\int_{V}d^{3}r^{\prime }\;L\left(r,r^{\prime }\right)c\left(r^{\prime },t\right)\; \end{array}$$

In scalar-product form, the righthand side is (α,Lc), where L is a linear integral operator with kernel *L*(***r***, ***r***′) (see [11], Eq (59)).

If we again assume that the drug sensitivity is independent of time during the therapy, we can retrace the steps to (16) and write

$$\begin{array}{c} \psi_{Y|c}\left(\xi \right)=\Psi_{\alpha |c}\left\{-\xi \left[L\;\text{AUC}\right]\left(r,t_{0}\right)\right\}\;. \end{array}$$ (21)

where $\left[L\;\text{AUC}\right]\left(r,t_{0}\right)=\int_{V}d^{3}r^{\prime }\;L\left(r,r^{\prime }\right)AUC\left(r^{\prime },t_{0}\right)$.

#### Drug distribution at the microscale

So far we have considered the drug distribution *c*(***r***, *t*) within a tumor to be a general spatial random process that evolves in time. A more detailed treatment considers that the drug molecules may be in the capillaries, diffusing in the interstitial space, bound to surface receptors or internalized into the cytoplasm of malignant cells, so that the drug distribution can be decomposed as [49–52]

$$\begin{array}{c} c\left(r,t\right)=c^{cap}\left(r,t\right)+c^{diff}\left(r,t\right)+c^{bound}\left(r,t\right)+c^{int}\left(r,t\right)\;. \end{array}$$ (22)

Each term in this expansion is a spatial random process that evolves in time, and there are causal relations among them; e.g., the internalization process cannot happen before the binding, and the diffusion process begins with extravasation from the capillaries. There is also a process of intravasation where the drug can be returned to the circulation, but if this happens before binding and internalization it can be modeled as a simple reduction in extravasation rate. Most drugs are only effective when bound or internalized, so extracting estimates of these component concentrations will lead to better prediction of efficacy. The models presented below, which assume dynamic equilibrium, relate the four components to each other in such a way that an estimate of one can be used to extract estimates of the subsequent processes.

While not explicitly modeled here, there is also the process of catabolism by which a drug molecule is broken down into smaller molecules, thereby losing its potency and disappearing from the effective drug distribution. Catabolism can occur during diffusion; within a tumor cell after internalization (where it is called intracellular catabolism), or along the excretion route for the drug, often in the kidney or liver. The time scale for catabolism ranges from a few hours to a few days.

#### Nonlocal processes with time delay: Diffusion

The first two terms in (22) are linked by the time-dependent diffusion equation for an inhomogeneous medium, given by [52]

$$\begin{array}{c} \frac{\partial }{\partial t}c^{diff}\left(r,t\right)-\nabla \cdot \left[D\left(r\right)\nabla c^{diff}\left(r,t\right)\right]=s\left(r,t\right)\;, \end{array}$$ (23)

where *D*(***r***) is the diffusion coefficient and *s*(***r***, *t*) is the source of the diffusing species (e.g. drug molecules). For our purposes we will treat both *D*(***r***) and *s*(***r***, *t*) as random processes, and the initial condition *c*^*diff*^(***r***, 0) is taken to be zero.

In many cases drug molecules escape from tumor capillaries through small pores called fenestrations, typically 60-80 nm diameter. Most drug molecules are a few nm across, so they can easily escape from the capillaries, as can antibodies, which are 10-15 nm. For comparison, a water molecule is about 0.2 nm and a red blood cell is 8,000 nm (8 *μ*m).

Because the pores are small compared to other relevant dimensions in a tumor, they can be modeled as Dirac delta distributions, and one sample of the random process *s*(***r***, *t*) is given by

$$\begin{array}{c} s\left(r,t\right)=\sum_{j=1}^{J}\delta \left(r-r_{j}\right)\;\delta \left(t-t_{j}\right)\;, \end{array}$$ (24)

where the *j*^*th*^ drug molecule (*j* = 1, …, *J*) escapes into the extravascular space at point ***r*** = ***r***_*j*_ and time *t* = *t*_*j*_, There are 2*J* + 1 random quantities in this expression: *J* 3D position vectors ***r***_*j*_, *J* times *t*_*j*_, and *J* itself. The units of *s*(***r***, *t*) are drug molecules per unit volume per unit time.

If there is no randomness other than these 2*J* + 1 variables and the molecules are secreted independently, then *s*(***r***, *t*) is a Poisson point process (see [10] (Chapter 11), [11, 53] and S2 Appendix). The characteristic functional of a spatiotemporal Poisson point process is fully determined by its mean function $\bar{s}\left(r,t\right)$:

$$\begin{array}{c} \Psi_{s|\bar{s}}\left[\phi \right]=\exp\left[\int_{V}d^{3}r\int_{0}^{T}dt\;\bar{s}\left(r,t\right)\left(e^{-2\pi i\phi (r,t)}-1\right)\right]\;, \end{array}$$ (25)

where *T* is the time interval over which the delta functions can occur. The notation $\Psi_{s|\bar{s}}\left[\phi \right]$ indicates that $\bar{s}\left(r,t\right)$ is held constant in the average over the sets {***r***_*j*_} and {*t*_*j*_}. Note that (25) is now a *spatiotemporal* characteristic functional, as opposed to (1). This illustrates the fact that the individual molecular emission events in (24) occur at random times; this fine-scale behavior is effectively unobservable *in vivo*, but we will use (25) to derive a spatial random process, whose characteristic functional is of the form (1), that is observable.

Physiologically, $\bar{s}\left(r,t\right)$ depends on the concentration of the drug in the capillaries *c*^*cap*^(***r***, *t*) and the vascular permeability *v*(***r***). If there are no nonlinearities in the secretion process, this dependence is a simple product, $\bar{s}\left(r,t\right)=c^{cap}\left(r,t\right)v\left(r\right)$, and we can write

$$\begin{array}{c} \Psi_{s|c^{cap},v}\left[\phi \right]=\exp\left[\int_{V}d^{3}r\int_{0}^{T}dt\;c^{cap}\left(r,t\right)v\left(r\right)\left(e^{-2\pi i\phi (r,t)}-1\right)\right]\;. \end{array}$$ (26)

If ***c***^*cap*^ and **v** are also treated as random processes, we must average over them to obtain the overall characteristic functional for the source term:

$$\begin{array}{c} \Psi_{s}\left[\phi \right]={\left\langle {\left\langle \exp\left[\int_{V}d^{3}r\int_{0}^{T}dt\;c^{cap}\left(r,t\right)v\left(r\right)\left(e^{-2\pi i\phi (r,t)}-1\right)\right]\right\rangle }_{c^{cap}}\right\rangle }_{v}\;. \end{array}$$ (27)

These two averages can be approximated with sample averages if one has a constructive model for the tumor capillaries (e.g., the fractal model of Baish and Jain [54] or the Anderson-Chaplain model [55]).

We can write the diffusion equation above in vector-space form as

$$\begin{array}{c} \frac{\partial c^{diff}\left(t\right)}{\partial t}+Dc^{diff}\left(t\right)=s\left(t\right)\;, \end{array}$$ (28)

where

$$\begin{array}{c} D\equiv -\nabla \cdot D\nabla \;. \end{array}$$ (29)

It is shown in S4 Appendix that the characteristic functional for the diffusing component of the drug is related to the characteristic functional of the source term by

$$\begin{array}{c} \Psi_{c^{diff}}\left[\phi ,t\right]={\left\langle \Psi_{s}\left[\int_{0}^{t}dt^{\prime }\exp\left[\left(t^{\prime }-t\right)D^{†}\right]\phi \right]\right\rangle }_{D}\;. \end{array}$$ (30)

Explicit forms for the diffusion operator D and its adjoint $D^{†}$ are also given in S4 Appendix. It is shown there that the diffusion process functions as a low-pass filter, rapidly suppressing fine details in *c*^*diff*^(***r***, *t*) as the drug diffuses farther from its source.

#### Binding to receptors in targeted therapy

Binding of a ligand to a receptor is conventionally parameterized by the receptor concentration *B*_*max*_, the dissociation constant *K*_*d*_ and the binding potential *BP* = *B*_*max*_/*K*_*d*_ [56]. At tracer levels *BP* is the ratio of specifically bound ligand concentration to free concentration. In the usual approach where the concentrations are not considered to vary with position and are not treated as random, the equilibrium concentration of bound ligands is given by the Michaelis-Menten equation, a nonlinear relation given by

$$\begin{array}{c} C^{bound}=B_{max}\frac{C^{free}}{K_{d}+C^{free}}\;. \end{array}$$ (31)

To adapt this result to the formalism of this paper, we interpret the receptor density *B*_*max*_ as the random density of tumor cells *n*(***r***, *t*) times the random number of receptors per cell, *N*_*rec*_, and we can identify *C*^*free*^ as the random process *c*^*diff*^(***r***, *t*). If we assume that *c*^*diff*^(***r***, *t*) varies sufficiently slowly that dynamic equilibrium is maintained at each point, we can write:

$$\begin{array}{c} c^{bound}\left(r,t\right)=n\left(r,t\right)N_{rec}\frac{c^{diff}\left(r,t\right)}{K_{d}+c^{diff}\left(r,t\right)}\;. \end{array}$$ (32)

Because tumor cells are genetically very similar (probably all clones of a single parent cell), a reasonable statistical model for the number of receptors per cell is that it is a Poisson random variable with mean ${\bar{N}}_{rec}$, which is independent of ***r***. It may also be valid to assume that *K*_*d*_ is the same for all cancer cells with receptors for a particular targeted drug. In that case *K*_*d*_ can be taken as nonrandom and independent of ***r***.

In clinical practice it is useful to maintain *c*^*diff*^(***r***, *t*) ≪ *K*_*d*_ so that the binding potential is small; otherwise the drug molecules may all be bound on a thin layer of cells at the periphery of a large tumor, leaving few to treat malignant cells in the interior; Weinstein at al. refer to this effect as the binding-site barrier [57]. It is avoided in the weak-binding limit, where we linearize (32) about zero to write

$$\begin{array}{c} c^{bound}\left(r,t\right)\approx \frac{n\left(r,t\right)N_{rec}}{K_{d}}c^{diff}\left(r,t\right)\;. \end{array}$$ (33)

With this approximation, ***c***^*bound*^ is proportional to ***c***^*diff*^, which in turn is a linear transform of the source **s** in the diffusion equation. The corresponding characteristic functional is

$$\begin{array}{c} \Psi_{c^{bound}}\left[\phi \right]={\left\langle \Psi_{c^{diff}}\left[\frac{N_{rec}}{K_{d}}n\phi \right]\right\rangle }_{N_{rec},n} \end{array}$$ (34)

If the individual fenestrations in the capillaries secrete drug molecules independently into the tumor, it follows from the central-limit theorem that ***c***^*diff*^ is a Gaussian random process. Because a linear transformation of a Gaussian random process is also Gaussian, it then follows in this linearized model that Ψ_***c***^*bound*^_(***ϕ***) is the characteristic functional for a Gaussian random process, with a known analytical form (see S2 Appendix), but in general with unknown mean function and covariance operator.

#### Internalization

A general term for internalization is endocytosis. Specific mechanisms include phagocytosis, pinocytosis, receptor-mediated endocytosis and clathrin-mediated endocytosis. For a review, see [58].

A simple model of endocytosis presented by Wiley in 1982 [59] defines an endocytotic rate constant *K*_*e*_ through the equation (in our notation),

$$\begin{array}{c} \frac{dC^{int}}{dt}=K_{e}C^{bound}\;. \end{array}$$ (35)

If we replace the overall concentrations with the corresponding random processes and integrate over time, assuming a chemotherapy administration at *t* = 0, we obtain

$$\begin{array}{c} c^{int}\left(r,t\right)=K_{e}\int_{0}^{t}dt^{\prime }c^{bound}\left(r,t^{\prime }\right)\;. \end{array}$$ (36)

With (32), we have

$$\begin{array}{c} c^{int}\left(r,t\right)=K_{e}N_{rec}\int_{0}^{t}dt^{\prime }n\left(r,t^{\prime }\right)\frac{c^{diff}\left(r,t^{\prime }\right)}{K_{d}+c^{diff}\left(r,t^{\prime }\right)}\;. \end{array}$$ (37)

The events that occur after internalization of a cytotoxic drug are complex, and mathematical models are largely lacking. For a qualitative review, see [60].

## Application to precision cancer therapy

### Approach

To this point we have derived characteristic functionals for a number of physiological random processes related to cancer therapy, and we have used the results to obtain finite-dimensional characteristic functions and probability density functions for clinically relevant random variables such as the integrated log kill, which then permits us to compute probability of tumor control as in Eqs (18)–(20). All of these quantities are defined in terms of hypothetical infinite ensembles of patients, so their use for individuals is so far somewhat limited. In precision medicine, the objective is to make use of patient-specific data and mathematical models to enhance decision making; refer again to Fig 1. This process begins with identifying clinically relevant treatment efficacy biomarkers such as the integrated log-kill *Y*; because *Y* may not be directly observable, we must also identify relevant clinically observable physiological processes; we have discussed several spatiotemporal processes which are accessible via molecular imaging. Then, by combining imaging data and the mathematical models presented here, we can compute an estimate of *Y* which is specific to a particular patient. Even with patient-specific imaging, uncertainties remain, and thus the patient-specific *Y* is still a random variable, but these uncertainties are almost certainly lower than if no imaging were performed. We discuss the statistical properties of patient-specific estimates of *Y* in a later section (Eqs (39)–(43) and Discussion).

In several previous papers [9, 52, 61–63], we have considered the problem of *optimizing* a diagnostic imaging study or therapeutic regimen in order to get the best outcome for an individual patient. This would seem to be fundamentally impossible since both diagnostic and therapeutic efficacy are typically defined in terms of large groups of patients and evaluated by clinical trials, but we have developed rigorous mathematical methods for patient-specific optimization of SPECT imaging [9], radiation therapy [61, 62] and chemotherapy [52, 63]. None of these papers, however, makes use of random processes and characteristic functionals as in this paper.

In the paradigm of personalized medicine it is reasonable to expect most cancer patients to undergo genomic analysis and in many cases to have sensitivity-resistance assays to aid in selection of therapeutic drugs. In addition, there are attempts to use databases to correlate genomic mutations with drug efficacy in the hope that genomically similar patients will have similar drug responses. However this effort is problematic because there is no consensus on how to define genomic similarity and because a genomic mutation leads to an abnormal protein only after transcription and translation. For this reason there is considerable interest in the transcriptome and the proteome, but the databases are not as extensive at these levels.

The paradigm we consider for personalized cancer therapy is thus [52, 64]:

1. Obtain images, genomic data and other data for a specific patient;
2. Optimize the therapy regimen using this information and supporting mathematical models;
3. Evaluate the resulting patient-specific protocol with multiple patients as in conventional clinical trials.

The first step requires reconstruction of molecular images for each patient, which requires the application of statistical image science methodologies [10]; the spatial statistics of reconstructed images are discussed in [10, 65] and elsewhere.

The second step requires the application of mathematical or heuristic models for estimating the probability of tumor control and probability of normal tissue complication for each patient, and all of the expressions for Pr(*TC*) given in this paper are potentially applicable. To optimize a treatment, we advocate using a curve-based analysis such as the Therapy Operating Characteristic (TOC) curve discussed below and elsewhere [52], which models the trade-off between tumor control and undesired side effects.

The third step requires recruiting multiple patients and applying the joint data collection and adaptive therapy protocol independently to each subject in the trial.

**Fig 5:**
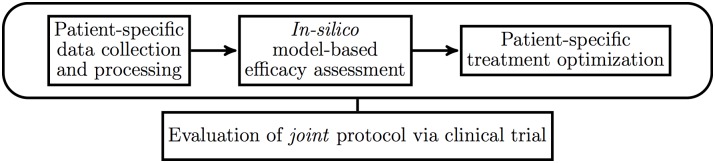
Precision medicine trials. Our proposed clinical trial framework for precision medicine consists of selecting a data collection, processing and model-based decision-making protocol, then testing the entire joint protocol in a classical trial setting.

This paradigm provides a logical structure for clinical trials of personalized therapy, yielding a joint evaluation of the quality of the patient-specific data; the strategy for adapting the therapy regimen to the patient, and the models that connect the data and the strategy. As we stressed in [52], the only thing one can ever evaluate in a clinical trial for personalized medicine is the combination of the patient-specific data, the model of tumor response and the personalization strategy (see Fig 5).

### Therapeutic efficacy

**Fig 6:**
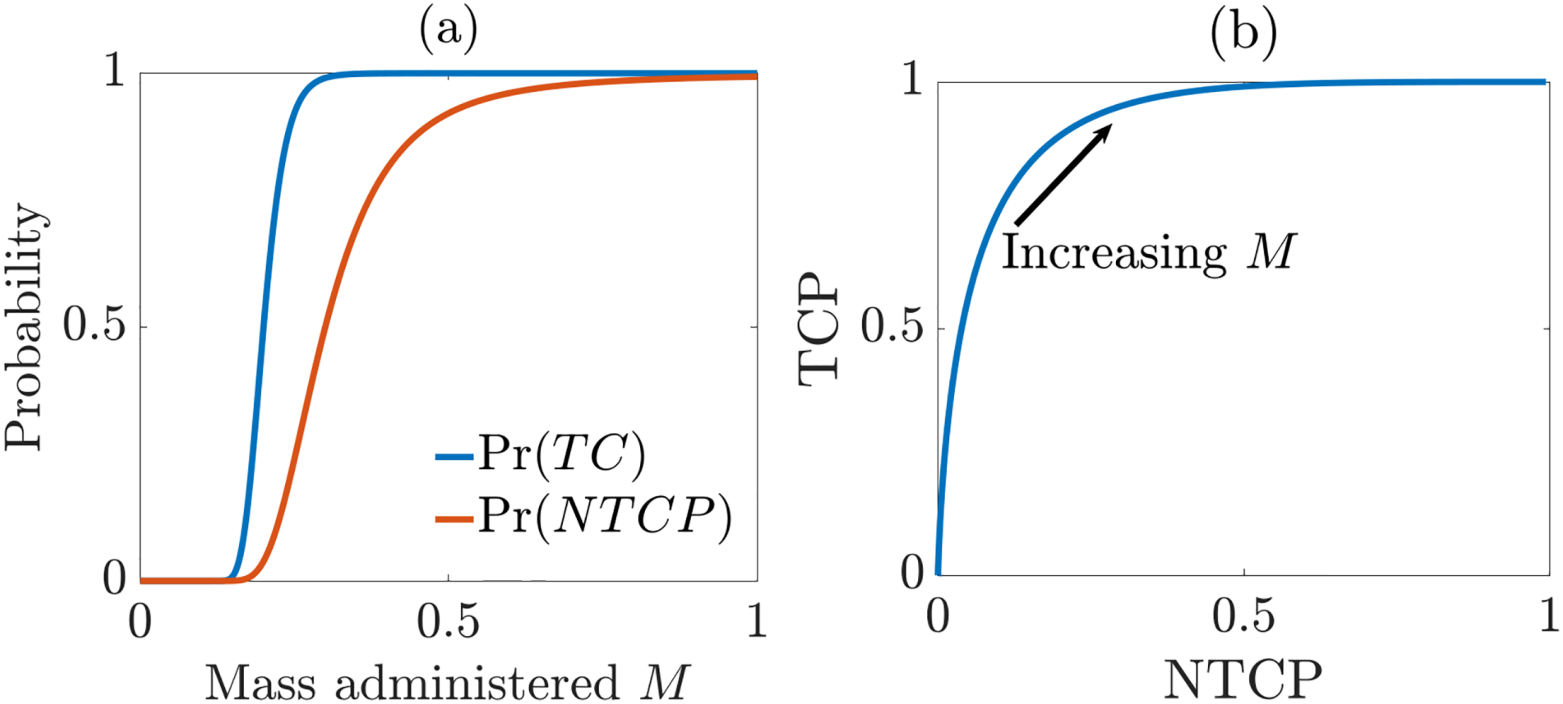
TOC curves. An illustration of a Therapy Operating Characteristic (TOC) curve. (a) Schematic plot of Tumor-Control Probability (TCP) and Normal-Tissue-Complication Probability (NTCP) vs. injected mass of drug, *M* (arbitrary units); (b) The TOC curve: Plot of TCP vs. NTCP as *M* is varied.

Any optimization procedure requires the selection of a scalar figure of merit to maximize or minimize, so an important question in the second step of the precision medicine paradigm proposed above is the selection of an appropriate figure of merit. One could imagine maximizing probability of tumor control, Pr(*TC*), over an admissible set of therapy parameters such as administered mass, but tumor control by itself does not constitute therapeutic success; we must also be concerned with adverse side effects. Both the tumor-control probability (TCP) and the probability of some specified normal-tissue complication (NTCP) generally increase with drug dosage. If the total mass of the drug administered to patient *j* is denoted *M*, then with suitable models such as those given in Eqs (18)–(20), we can calculate TCP_*j*_(*M*) and NTCP_*j*_(*M*) for patient *j* and plot both as a function of *M* as shown in Fig 6(a). By plotting TCP_*j*_(*M*) vs. NTCP_*j*_(*M*) as the drug mass is varied, we thus construct the patient-specific Therapy Operating Characteristic (TOC) curve, as shown in Fig 6(b), which is the therapeutic analog of the familiar Receiver Operating Characteristic (ROC) curve, used routinely in evaluating diagnostic imaging methods and laboratory tests. The TOC curve was originally developed in the context of radiation therapy [61, 62] and recently extended to chemotherapy [52]. Ultimately, we expect the TOC curve to be a useful, simplified graphical decision-making tool in clinical practice; the clinician and patient can together select treatment parameters that balance the chance of success and chance of side effects according to the patient’s level of risk aversion.

As with ROC curves, we can use the area under the TOC curve, denoted AUTOC, as a metric of therapeutic efficacy and hence as a measure of both treatment quality and image quality for situations where the images are used for therapy planning. This approach is particularly attractive for radiation therapy where established radiobiological models can be used to compute TCP and NTCP, and the theory developed in this paper facilitates the estimation of TCP in chemotherapy. Estimation of NTCP in chemotherapy is more problematic, in large part because there is very little literature on the whole-body distributions of common drugs and their patient-to-patient variability. One can find publications on maximum tolerated dose (MTD) and the corresponding dose-limiting side effect, but very little on the variation of NTCP with injected mass of the drug. Moreover, the injected mass is always reported as milligrams per square meter of body surface area (possibly also per week).

### Estimation tasks in precision medicine

#### Estimation of patient-specific parameters

Information about the drug distribution for a particular patient can be obtained by using radiolabelled drug molecules that can be imaged with PET or SPECT. The sensitivity of these imaging modalities is high, so the drug can probably be administered at subtherapeutic levels, but if this is a worry, then a surrogate molecule with approximately the same molecular weight as the drug but with no therapeutic effect can be used to study the extravasation of the drug from the capillaries (the source term in the diffusion Eq (23)) and its diffusion through the extracellular space. For a bolus injection the extravasation and diffusion processes have very different time dependences (the former being more rapid), so in principle we can separate *c*^*cap*^(***r***, *t*) from *c*^*diff*^(***r***, *t*) on the basis of dynamic PET or SPECT images, for either the actual drug or the surrogate.

Assume we have used a radiolabeled drug with long half life to observe the overall drug concentration *c*_*j*_(***r***, *t*) for patient *j* over a long time period *T*. As mentioned in the introduction, imaging data ***g*** and the radiolabeled drug ***c*** form a pair of statistical covariates {***c***, ***g***}. For patient *j*, the raw imaging data ***g***_*j*_ = ***g***|***c***_*j*_ is a statistical quantity whose distribution is *indirectly* related to ***c***_*j*_ via a linear operator $H_{c}:L^{2}\left(V\right)\to R^{M}$, which maps the function ***c***_*j*_ to a finite list of *M* average pixel counts. Specifically, many molecular imaging systems result in data that are accurately modeled as an *M*-dimensional Poisson random vector with mean given by $H_{c}c_{j}$, i.e. if *g*_*m*_ denotes the *m*th pixel data, we have [10]

$$\begin{array}{c} \text{Pr}\left(g|c_{j}\right)=\prod_{m=1}^{M}\frac{{\left[{\left(H_{c}c_{j}\right)}_{m}\right]}^{g_{m}}}{g_{m}!}\exp\left(-{\left(H_{c}c_{j}\right)}_{m}\right) \end{array}$$ (38)

The raw image data for patient *j* are then a random sample from Pr(***g***|***c***_*j*_), from which one can perform statistical estimation to *reconstruct* ***c***_*j*_; such an estimate is denoted generically by a hat. In molecular imaging, the most common reconstruction methods are variants on classical Maximum Likelihood (ML) estimation, that is we choose ${\hat{c}}_{j}$ to be (at least approximately) a maximizer of Pr(***g***|***c***_*j*_) [10].

Suppose further that we have used some tracer to measure drug sensitivity *α*(***r***). The data for this study are denoted ***g***|***α***_*j*_, and the linear operator for this step is denoted $H_{\alpha }$. We can use the data sets ***g***|***c***_*j*_ and ***g***|***α***_*j*_ to perform ML reconstructions of the drug distribution and sensitivity, denoting the results as results as ${\hat{c}}_{j}$ and ${\hat{\alpha }}_{j}$, respectively. Note that both ${\hat{c}}_{j}$ and ${\hat{\alpha }}_{j}$ can now be considered as *patient-specific* physiological random processes; the randomness now is a result of the noise in the imaging system (which is well characterized for emission imaging). The statistical properties of ${\hat{c}}_{j}$ and ${\hat{\alpha }}_{j}$ are related to the estimation procedure used to construct them. In particular, if Maximum Likelihood estimation with the iterative EM algorithm is employed [66, 67], we can use a combination of asymptotic statistics and Monte Carlo simulation to compute the properties of these estimates [65]. In particular, in the appropriate asymptotic regime, ${\hat{c}}_{j}$ and ${\hat{\alpha }}_{j}$ can both be accurately modeled as Gaussian random processes. Note that Gaussianity in this context is a result of asymptotic statistical properties and does not depend on any linearity properties of the imaging system or the EM reconstruction algorithm (which is itself a nonlinear iterative algorithm). Systematic errors, due usually to the presence of a nontrivial null space or rapidly decaying singular spectrum of the imaging operators $H_{c}$ and $H_{\alpha }$, or the presence of nuisance parameters such as a nonzero attenuation field, may also be present and must be accounted for in the statistical model for ${\hat{c}}_{j}$ and ${\hat{\alpha }}_{j}$, which in these instance will most likely be non-Gaussian. Such errors typically manifest as reconstruction artifacts or degraded resolution in the reconstructed image. In this case, the exact statistical properties of ${\hat{c}}_{j}$ and ${\hat{\alpha }}_{j}$ may be difficult to derive analytically, and it may be necessary to apply Bayesian techniques to compute probabilities with ${\hat{c}}_{j}$ and ${\hat{\alpha }}_{j}$.

Referring again to Fig 2, we will now employ the proposed treatment response model (Eq (13)) and the patient-specific PRPs ${\hat{c}}_{j}$ and ${\hat{\alpha }}_{j}$ in place of the generic PRPs ***c*** and ***α*** in order to personalize the parameter *Y* and hence the probability of tumor control Pr(*TC*). Plugging ${\hat{\alpha }}_{j}$ and ${\hat{c}}_{j}$ into (13), we obtain

$$\begin{array}{c} {\hat{Y}}_{j}=-\int_{V}d^{3}r\;{\hat{\alpha }}_{j}\left(r\right)\;{\hat{AUC}}_{j}\left(r\right) \end{array}$$ (39)

where ${\hat{AUC}}_{j}\left(r\right)$ is the area under the curve of the estimated drug concentration at point ***r*** vs. time:

$$\begin{array}{c} {\hat{AUC}}_{j}\left(r\right)\equiv \int_{t_{0}}^{t_{0}+T}dt\;{\hat{c}}_{j}\left(r,t\right)\;. \end{array}$$ (40)

Note that as (39) is a functional of an approximate maximum likelihood estimate, it is guaranteed to be asymptotically normal by an application of the (functional) delta method; see [68, 69] for a technical discussion. One can also interpret (39) as an application of the maximum likelihood invariance theorem [10], but the pair of functions {***c***, ***α***} must be considered infinite-dimensional nuisance parameters in this context, and so the definition of the likelihood for the parameter *Y* requires care [68].

To estimate patient-specific Pr(*TC*) using the definition Pr(*TC*) = Pr(*Y* < *Y*_*c*_), we require the PDF of the scalar random variable ${\hat{Y}}_{j}$; as just discussed, this PDF is approximately Gaussian. If we can assume that ${\hat{\alpha }}_{j}$ and ${\hat{c}}_{j}$ are unbiased estimates of ***α***_*j*_ and ***c***_*j*_, the mean of ${\hat{Y}}_{j}$ is the true value *Y*_*j*_, that is,

$$\begin{array}{c} {\left\langle {\hat{Y}}_{j}\right\rangle }_{{\hat{Y}}_{j}|\left\{c_{j},\alpha_{j}\right\}}=Y_{j}=-\int_{V}d^{3}r\;\alpha_{j}\left(r\right)AUC_{j}\left(r\right) \end{array}$$ (41)

In current practice, ${\hat{\alpha }}_{j}$ and ${\hat{c}}_{j}$ will usually be obtained by an iterative algorithm called MLEM (Maximum-Likelihood Expectation Maximization), which returns voxelized approximations to the estimates of the functions *α*_*j*_(***r***) and *c*_*j*_(***r***, *t*), respectively. The image data will also be sampled in time with a resolution Δ*t*.

With these interpretations and the properties of ML estimators (including the statistical independence of the two ECT data sets), we can show that the variance of ${\hat{Y}}_{j}$ is approximately given by

$$\begin{array}{c} \text{Var}\left({\hat{Y}}_{j}\right)\approx \epsilon^{3}\Delta t\sum_{n,n^{\prime }}\sum_{m,m^{\prime }}K_{{\hat{\alpha }}_{j}|\alpha_{j}}\left(r_{n},r_{n^{\prime }}\right)K_{{\hat{c}}_{j}|c_{j}}\left(r_{n},t_{m};r_{n^{\prime }},t_{m^{\prime }}\right), \end{array}$$ (42)

where *ϵ*^3^ is the volume of a voxel, Δ*t* is the time between images in a dynamic sequence, and $K_{{\hat{\alpha }}_{j}|\alpha_{j}}$ and $K_{{\hat{c}}_{j}|c_{j}}\left(r_{n},t_{m};r_{n^{\prime }},t_{m^{\prime }}\right)$ are elements of the covariance matrices for the estimates of the drug sensitivity and drug concentration, respectively. An efficient algorithm for computing these covariance matrices is given in [65].

We can thus assume that ${\hat{Y}}_{j}$ is approximately normal with mean (41) and variance (42), meaning that an estimate of the tumor control probability for patient *j* is given by

$$\begin{array}{c} \hat{TCP_{j}}=\int_{-\infty }^{Y_{c}}d{\hat{Y}}_{j}\;\text{pr}\left({\hat{Y}}_{j}\right)\;, \end{array}$$ (43)

Note that the mean and variance of ${\hat{Y}}_{j}$ are unknown; their values may be replaced by their sample equivalents, then confidence intervals constructed by standard means.

With (42) and (43), the estimated TCP for patient *j* is an error function as in [52].

#### Estimation of ensemble statistics

Now suppose we have ECT data for ***c***_*j*_, and hence a maximum-likelihood reconstruction ${\hat{c}}_{j}$, but no patient-specific ECT data for ***α***_*j*_ are available. In this case we can still use (39) with ***c*** replaced by ${\hat{c}}_{j}$, but we must now treat ***α*** as a nuisance parameter, i.e. one that influences the task but is not estimated. As discussed in [10], Sec. 13.3.8, the best approach is to marginalize the expression for TCP over ***α***, which requires a random process model for ***α***. When possible, it is preferable to choose a model based on appropriate training data, i.e. we estimate a characteristic functional **Ψ**_***α***_ by employing data collected from some other non-patient-specific means. A methodology for estimating characteristic functionals from training data is given by Kupinski et al. [12]; another class of methods for generating random field models from training image data is based on the maximum entropy principle and was first discussed in Zhu, Wu and Mumford [70]. Note that without patient-specific imaging data for a process such as ***α***, the uncertainty in the prediction of treatment response will certainly be greater.

## Discussion

The basic tools of physiological random processes, characteristic functionals, emission computed tomography and TOC curves, in combination, could lead to a wide variety of new applications in basic research and clinical medicine, especially in oncology.

One way to explore possible new applications is to return to the hallmarks of cancer, as enumerated in the Introduction, and to realize that each hallmark is driven by multiple interacting physiological random processes (PRPs). Each PRP in turn is associated with the expression of multiple genes and production of multiple proteins. With the recent advances in biomolecular engineering, it is reasonable to expect that ECT probes that bind to virtually any of these proteins can be developed. There should also be considerable freedom regarding which ECT modality to use (PET, SPECT, CPET or OpET; see Introduction). Often we will be able to use two or more ECT modalities simultaneously and to use the energy or wavelength variable to distinguish multiple probes in each modality. Then, the theory of interacting random processes can be used to provide a comprehensive statistical characterization of multiple proteins associated with a given hallmark or pathway.

It is this ability to characterize multiple PRPs quantitatively and simultaneously and to use models to predict treatment efficacy that sets the approach in this paper apart from quantitative imaging biomarkers (QIBs). In broad general terms, a QIB is any numerical value derived from an image that is intended to be indicative of physiology or pathology in the patient being imaged. More specifically, the goal of current research in this area, as stated in [71], is to produce “standardized, unbiased, and precise imaging data in support of the larger medical research and clinical enterprise.” In a sense, we are proposing to expand the reach of QIBs to include biomarkers which are not directly measurable from imaging data (such as tumor volume or standardized uptake values), but which may require an auxiliary biomathematical model to compute.

While we have considered explicitly the case of monotherapy in this work, the PRP framework presented here naturally extends to combination therapies, with each drug comprising a separate PRP. When multiple drugs are to be employed, a mathematical efficacy model such as the integrated log-kill can be developed to predict response for the proposed combination therapy. Multiple ECT images—one corresponding to each PRP in the model—can then be collected to predict patient-specific response. Alternately, random process models can be employed if imaging data is unavailable for some of the PRPs involved. Furthermore, an extension of the TOC curve for multiple treatment parameters (such as dosage and timing for each drug) can be developed and the subsequent figure of merit maximized over the set of admissible treatment parameters. Theoretically, treatment optimization could take place in real-time as the actual treatment is administered, so long as longitudinal imaging data is available and the necessary efficacy models and corresponding figures of merit are rapidly computable. Complicated efficacy models may require, for example, the solution of a four-dimensional Partial Differential Equation (PDE), which may be computationally demanding, and the usage of expensive Monte Carlo simulation, which may prevent real-time adaptation. In contrast, the integrated log-kill model presented here only requires computing a dot product, and the explicit probability densities we have derived bypass the need for computationally demanding Monte Carlo simulation.

Crucial to demonstable success with either PRPs or QIBs is validation. Validation of the PRP formalism in this paper may in fact be easier than validation of QIBs because we have specified the task we want to perform with PRPs. That task is to optimize a cancer treatment for an individual patient, with optimality defined by the area under a single-patient TOC curve. In that same section we outlined a methodology for performing clinical trials with such personalized therapy, and we noted that all one can ever evaluate in this scenario is the combination of the patient-specific data, the model of tumor response (and how it varies with administered mass of the drug) and the personalization strategy. The final metric for success in this scenario of then some measure of therapeutic efficacy for a cohort of patients; ultimately, conventional survival curves can be used.

With this viewpoint, optimization of the strategy reduces to getting better patient data, developing better models of therapeutic efficacy and developing correspondingly better personalization strategies. Each of these goals is an area for future research, and each fits within the theory introduced in this paper.

## Conclusion

This paper has presented an approach to modeling physiological processes using random functions, or equivalently as random infinite-dimensional vectors. The statistical properties of these random functions are described fully by the key tool used in this paper, the characteristic functional. Moreover, conditional and joint characteristic functionals provide rigorous new methods for describing the linear and nonlinear interactions among random processes. We have illustrated the power of these approaches by developing analytic formulas for various situations of interest in oncology.

Many of the explicit results in this paper are based on the linear-log-kill expression (14), which has the advantage of immediately reducing two key infinite-dimensional random processes to a single scalar, the integrated log kill. Tumor response to a drug is more complicated than implied by this equation, so we have also considered nonlinear and nonlocal effects in tumor response. In all cases it was possible to derive analytic forms for the statistics of the integrated log kill *Y*. Nevertheless, there is still a need for auxiliary studies in order to select which additional effects should be incorporated into the theory, how they should be integrated and how any relevant patient-specific parameters can be estimated.

We have also shown that results relevant to personalized cancer therapy can be derived if we have patient-specific molecular imaging data on the particular physiological processes that affect the growth of that patient’s tumor and its response to therapy. The key mathematical tools in this endeavor were the properties of maximum-likelihood estimates and the Therapy Operating Characteristic (TOC) curve.

## Supporting information

S1 Appendix Mathematical properties of characteristic functionals. (PDF)

S2 Appendix Example random process models. (PDF)

S3 Appendix Gompertzian tumor growth as a random process. (PDF)

S4 Appendix Characteristic functionals for diffusion. (PDF)
