# Supplementary material for: Physiological random processes in precision cancer therapy
Source: PLoS One. 2018 Jun 29;13(6):e0199823. doi: 10.1371/journal.pone.0199823 (PMC6025881; doi:10.1371/journal.pone.0199823)
Supplement: S1 Appendix — (PDF) [file pone.0199823.s001.pdf]

S1 Appendix: Mathematical Properties of Random Processes

We have considered several spatiotemporal physiological quantities in this work, such as the drug concentration $c(\boldsymbol{r}, t)$, susceptibility $\alpha(\boldsymbol{r})$, and diffusion coefficient $D(\boldsymbol{r})$. The point of view of this work is that such physiological processes can be modeled as random spatiotemporal functions $\boldsymbol{f} = f(\boldsymbol{r}, t)$. This assumption facilitated the rigorous computation of clinically relevant quantities such as probability of tumor control.

The goal of this supplement is to provide a brief discussion of some of the mathematical properties of random processes that were used in this work. General references for spatial random processes include [1, 2]; for an introduction in the context of image science, see [3, 4].

The statistical properties of a random quantity $\boldsymbol{X}$ can be quantified by defining a probability function for $\boldsymbol{X}$, which assigns a number $0 \leq \Pr(\boldsymbol{X} \in E) \leq 1$ that $\boldsymbol{X}$ assumes values in the event set E. This can either be interpreted as a limiting frequency of occurrence in repeated trials, or as a prediction of chance conditional on available information [5]. Both interpretations are useful in the context of precision medicine.

We have assumed in this work that all finite-dimensional random quantities posses a *Probability Density Function* or *PDF*, which is a (possibly generalized) function $\mathrm{pr}(\boldsymbol{x})$ defined so that the average of any function of $\boldsymbol{X}$ is given by

$$\big\langle g(\boldsymbol{X}) \big\rangle = \int_{\infty} d^n x \; g(\boldsymbol{x}) \mathrm{pr}(\boldsymbol{x}), \tag{1}$$

where n is the dimension of $\boldsymbol{X}$. As a special case, the probability $\Pr(\boldsymbol{X} \in E)$ is given by (1) with $g(\boldsymbol{x}) = 1$ if $\boldsymbol{x} \in E$ and zero otherwise. In this sense, the PDF $\mathrm{pr}(\boldsymbol{x})$ contains all statistical information about $\boldsymbol{X}$, because any desired average value or probability can be obtained from it. If $\boldsymbol{X}$ is a random process, however, the issue of defining a PDF is much more subtle. Assuming that realizations are square integrable functions, i.e. $\boldsymbol{f} \in L^2$, we would austensibly require a PDF in infinitely many variables as the dimension of L^2 is infinite. While it is still sometimes possible to define PDFs for random processes, doing so requires fixing a so-called *reference measure*, and the choice of reference measure is usually not clear in infinite dimensions [6]. We avoid this technicality by working with the characteristic functional.

As stated in the main text, the spatial statistics of a random process $f(\boldsymbol{r}, t)$ are fully described by the characteristic functional, defined as

$$\Psi_{\boldsymbol{f}}[\boldsymbol{\phi}, t] = \Big\langle \exp\left[-2\pi i (\boldsymbol{\phi}, \boldsymbol{f})\right] \Big\rangle \tag{2}$$

where the angle brackets indicate a statistical average (expectation) over all realizations of the process $\boldsymbol{f}$, and we define the scalar product of $\boldsymbol{\phi}$ and $\boldsymbol{f}$ as

$$(\boldsymbol{\phi}, \boldsymbol{f}) = \int_V d^3 r \; \phi(\boldsymbol{r}) f(\boldsymbol{r}, t). \tag{3}$$

We assume that the test function $\boldsymbol{\phi}$ is chosen so that (3) is well-defined; a more extensive discussion of this issue requires the theory of generalized functions which we avoid treating here [2, 4].

In Supporting Information S2, we provide several example functionals of the form (2). Note that (2) describes only the spatial statistics of $\boldsymbol{f}$; if joint statistics across multiple time points are required, we must re-define (2) and (3) so that the test function $\boldsymbol{\phi}$ depends on time. Supposing that $f(\boldsymbol{r}, t)$ is defined for $\boldsymbol{r} \in V$ and $t \geq 0$, we would have

$$\Psi_{\boldsymbol{f}}[\boldsymbol{\phi}] = \Big\langle \exp\left[-2\pi i(\boldsymbol{\phi}, \boldsymbol{f})\right] \Big\rangle, \quad (\boldsymbol{\phi}, \boldsymbol{f}) = \int_0^\infty dt \int_V d^3r \; \phi(\boldsymbol{r}, t) f(\boldsymbol{r}, t).$$

The strategy outlined in this paper was to manipulate various random processes to derive a scalar random quantity Y. We then demonstrated how the statistics of Y (that is, the PDF of Y) could be derived from the relevant characteristic functionals. We will now describe how (2) can be used in general to derive useful statistical information about $\boldsymbol{f}$.

Extracting finite-dimensional statistical quantities

Given a random process $\boldsymbol{f}$, the statistics of any finite-dimensional quantities of interest can be obtained from (2). Immediately from the definition, we can obtain the characteristic *function* of any scalar product of the form $X = (\boldsymbol{\phi}, \boldsymbol{f})$, where $\boldsymbol{\phi}$ is a spatial test function, by noting that

$$\psi_X(\xi, t) = \Big\langle \exp[-2\pi i \xi X] \Big\rangle_X = \Big\langle \exp[-2\pi i \xi (\boldsymbol{\phi}, \boldsymbol{f})] \Big\rangle_{\boldsymbol{f}} = \Psi_{\boldsymbol{f}}[\xi \boldsymbol{\phi}, t]. \tag{4}$$

The PDF of X can then be obtained by inverse Fourier transform of $\psi_X(\xi)$. Similarly, given any finite dimensional vector of scalar products, say $\boldsymbol{X} = [(\boldsymbol{\phi}_1, \boldsymbol{f}), \ldots, (\boldsymbol{\phi}_n, \boldsymbol{f})]$, we can obtain the characteristic function of $\boldsymbol{X}$ by

$$\begin{aligned}
\psi_{\boldsymbol{X}}(\boldsymbol{\xi}, t) = \Big\langle \exp[-2\pi i \boldsymbol{\xi}^T \boldsymbol{X}] \Big\rangle &= \Big\langle \exp\left[-2\pi i \sum_{j=1}^n \xi_j (\boldsymbol{\phi}_j, \boldsymbol{f})\right] \Big\rangle \\
&= \Big\langle \exp\left[-2\pi i (\sum_{j=1}^n \xi_j \boldsymbol{\phi}_j, \boldsymbol{f})\right] \Big\rangle \\
&= \Psi_{\boldsymbol{f}}\left[\sum_{j=1}^n \xi_j \boldsymbol{\phi}_j, t\right].
\end{aligned} \tag{5}$$

The PDF of $\boldsymbol{X}$ can then be obtained by inverse Fourier transform of $\psi_{\boldsymbol{X}}(\boldsymbol{\xi}, t)$.

A special case of (5) occurs when we take $\boldsymbol{\phi}_j = \delta(\boldsymbol{r} - \boldsymbol{r}_j)$ for some collection of sample points $\boldsymbol{r}_1, \ldots, \boldsymbol{r}_n$. Then, (so long as $\Psi_{\boldsymbol{f}}$ is well-defined for such inputs),

the characteristic function of $\boldsymbol{X} = [f(\boldsymbol{r}_1, t), \ldots, f(\boldsymbol{r}_n, t)]$ would be given by

$$\psi_{\boldsymbol{X}}(\boldsymbol{\xi}, t) = \Psi_{\boldsymbol{f}} \left[\sum_{j=1}^{n} \xi_j \delta(\boldsymbol{r} - \boldsymbol{r}_j), t \right]$$

The statistics of nonlinear functionals of the process $\boldsymbol{f}$ can be obtained from $\Psi_{\boldsymbol{f}}$ by similar but more involved procedures. For instance, suppose a scalar Y is obtained by nonlinear transformation of a single scalar product, i.e. $Y = F[(\boldsymbol{\phi}, \boldsymbol{f})]$. The PDF of Y can be obtained by first using (4) to obtain the PDF of $(\boldsymbol{\phi}, \boldsymbol{f})$, then the PDF of Y is obtained via the standard PDF transformation law [4]. This can then be extended to the case where Y is a function of several scalar products using (5), and then the general case where $Y = F[\boldsymbol{f}]$ is a general nonlinear functional can be treated by expanding $\boldsymbol{f}$ in an orthonormal basis $\{\boldsymbol{e}_j\}$ and writing $Y = \tilde{F}[(\boldsymbol{f}, \boldsymbol{e}_1), (\boldsymbol{f}, \boldsymbol{e}_2), \ldots]$.

Joint and conditional characteristic functionals

Another strategy commonly employed in this paper was the usage of multiple interacting processes and *conditional* characteristic functionals. Given two random processes $\boldsymbol{f}$ and $\boldsymbol{g}$, we can consider their joint characteristic functional:

$$\begin{aligned} \Psi_{\boldsymbol{f},\boldsymbol{g}}[\boldsymbol{\phi}_1, \boldsymbol{\phi}_2, t] &= \Big\langle \exp\big[-2\pi i\big((\boldsymbol{\phi}_1, \boldsymbol{f}) + (\boldsymbol{\phi}_2, \boldsymbol{g})\big)\big] \Big\rangle \\ &= \Big\langle \exp\left[-2\pi i(\boldsymbol{\phi}_1, \boldsymbol{f})\right] \exp\left[-2\pi i(\boldsymbol{\phi}_2, \boldsymbol{g})\right] \Big\rangle. \end{aligned} \tag{6}$$

We say that $\boldsymbol{f}$ and $\boldsymbol{g}$ are *independent* if and only if (6) factors as the product (6) $= \Psi_{\boldsymbol{f}}[\boldsymbol{\phi}_1, t]\Psi_{\boldsymbol{g}}[\boldsymbol{\phi}_2, t]$. Fixing a realization of one or the other of $\boldsymbol{f}$ or $\boldsymbol{g}$ results in a *conditional* characteristic functional. For instance, fixing $\boldsymbol{g}$, the conditional characteristic functional of $\boldsymbol{f}|\boldsymbol{g}$ is defined as

$$\Psi_{\boldsymbol{f}|\boldsymbol{g}}[\boldsymbol{\phi}, t] = \Big\langle \exp[-2\pi i(\boldsymbol{\phi}, \boldsymbol{f})] \Big\rangle_{\boldsymbol{f}|\boldsymbol{g}}. \tag{7}$$

Note that (7) can be computed by first finding the joint PDF of the pair of random variables $X = (\boldsymbol{\phi}_1, \boldsymbol{f})$ and $Y = (\boldsymbol{\phi}_2, \boldsymbol{g})$, e.g. via the joint characteristic functional (6). Then, the conditional PDF for $X|Y$ can be derived, and (7) subsequently computed.

Given a conditional characteristic functional, we have the *chain rule* or *law of total expectation*, which states that

$$\Psi_{\boldsymbol{f}}[\boldsymbol{\phi}, t] = \Big\langle \Psi_{\boldsymbol{f}|\boldsymbol{g}}[\boldsymbol{\phi}, t] \Big\rangle_{\boldsymbol{g}} \tag{8}$$

Expressions similar to (4) and (5) can then be derived for functionals of multiple interacting random processes or conditional random processes by employing (6), (7) and (8); for example, if $Y = (\boldsymbol{f}, \boldsymbol{g})$ is the scalar product of two random processes, then the PDF of Y can be obtained by a combination of (8) and (3).

Moment functions

Another convenient description of the statistics of a random process comes via its moment functions. Given a process $\boldsymbol{f}$, the PDF of $f(\boldsymbol{r},t)$ is denoted $p_{\boldsymbol{r},t}(x)$ (note that this is the PDF of the one-point sample values of $f(\boldsymbol{r},t)$, not of the entire process). We then define the *mean function* of $\boldsymbol{f}$ as

$$\bar{\boldsymbol{f}} \equiv \bar{f}(\boldsymbol{r},t) = \langle f(\boldsymbol{r},t)\rangle = \int_{\mathbb{R}} dx\ x p_{\boldsymbol{r},t}(x)$$

Note that $\bar{f}(\boldsymbol{r},t)$ is a deterministic function: randomness has been 'averaged out'.

Now consider two samples, $f(\boldsymbol{r}_1,t)$ and $f(\boldsymbol{r}_2,t)$. If we compute their covariance, we arrive at the *covariance function*:

$$k_{\boldsymbol{f}}(\boldsymbol{r}_1,\boldsymbol{r}_2,t) = \mathrm{Cov}(f(\boldsymbol{r}_1,t), f(\boldsymbol{r}_2,t)) = \left\langle \big(f(\boldsymbol{r}_1,t) - \bar{f}(\boldsymbol{r}_1,t)\big)\big(f(\boldsymbol{r}_2,t) - \bar{f}(\boldsymbol{r}_2,t)\big)\right\rangle$$

The function $\boldsymbol{k_f}$ describes the second order correlation structure of any random process. For instance if $k_{\boldsymbol{f}}(\boldsymbol{r}_1,\boldsymbol{r}_2,t) = 0$, the values of the process at $\boldsymbol{r}_1, \boldsymbol{r}_2$ are uncorrelated; if $\bar{\boldsymbol{f}}$ is constant and $k_{\boldsymbol{f}}(\boldsymbol{r}_1,\boldsymbol{r}_2,t) \equiv k_{\boldsymbol{f}}(\boldsymbol{r}_1 - \boldsymbol{r}_2, t)$, then the second order statistics of $\boldsymbol{f}$ are shift-invariant and $\boldsymbol{f}$ is called is *wide-sense stationary* [1, 4]. We note that while the study of $\bar{\boldsymbol{f}}$ and $\boldsymbol{k_f}$ offers some useful insight into the structure of random processes, higher-order (i.e. three-point, four-point and so forth) correlation structures can be nontrivial, so in general we cannot assume that a process is completely described by its mean and covariance function; only the characteristic functional provides a complete description of $\boldsymbol{f}$ in general.

The moment functions of a random process can be recovered from the characteristic functional by taking certain functional derivatives; see [3, 7].

Transformation under a linear operator

One of the key features of the characteristic functional is that it behaves very favorably under linear transformation of realizations. Briefly, suppose that $\mathcal{A}$ is a bounded linear operator with adjoint $\mathcal{A}^\dagger$ (recall that $\mathcal{A}^\dagger$ is the unique operator such that $\big(\boldsymbol{\phi}, \mathcal{A}\boldsymbol{f}\big) = \big(\mathcal{A}^\dagger\boldsymbol{\phi}, \boldsymbol{f}\big)$ for all pairs $\{\boldsymbol{\phi}, \boldsymbol{f}\}$). Then, if we consider the process $g(\boldsymbol{r},t) = (\mathcal{A}f)(\boldsymbol{r},t)$, it is easy to see from the definition of $\Psi_{\boldsymbol{f}}$ that

$$\Psi_{\boldsymbol{g}}[\boldsymbol{\phi},t] = \left\langle \exp\left[-2\pi i\big(\boldsymbol{\phi}, \mathcal{A}\boldsymbol{f}\big)\right]\right\rangle = \left\langle \exp\left[-2\pi i\big(\mathcal{A}^\dagger\boldsymbol{\phi}, \boldsymbol{f}\big)\right]\right\rangle = \Psi_{\boldsymbol{f}}[\mathcal{A}^\dagger\boldsymbol{\phi},t] \quad (9)$$

References

[1] Christakos G. Random field models in earth sciences. Courier Corporation; 2012.

[2] Gel'fand IM, Vilenkin NY. Generalized functions, Vol. 4: applications of harmonic analysis. Academic Press; 1964.

[3] Clarkson E, Barrett HH. Characteristic functionals in imaging and image-quality assessment: tutorial. JOSA A. 2016;33(8):1464–1475.

[4] Barrett H, Myers K. Foundations of image science. John Wiley & Sons; 2004.

[5] Jaynes ET. Probability theory: the logic of science. Cambridge University Press; 2003.

[6] Sullivan TJ. Introduction to uncertainty quantification. Springer; 2015.

[7] Monin AS, Yaglom AM. Statistical fluid mechanics: mechanics of turbulence. vol. 1. Lumley JL, editor. The MIT Press; 1971.
