# Supplementary material for: Physiological random processes in precision cancer therapy
Source: PLoS One. 2018 Jun 29;13(6):e0199823. doi: 10.1371/journal.pone.0199823 (PMC6025881; doi:10.1371/journal.pone.0199823)
Supplement: S2 Appendix — (PDF) [file pone.0199823.s002.pdf]

S2 Appendix: Example Random Process Models

In this supplement, we offer a small collection of random processes models that are useful in modeling physiological and imaging systems, providing a functional form for the characteristic functional whenever possible. We display some numerical realizations, though we do not discuss simulation methods in depth; see e.g. the extensive recent book [1]. Refer also to the introductory paper [2].

Recall that the spatial characteristic functional is defined as

$$\Psi_{\boldsymbol{f}}[\boldsymbol{\phi}, t] = \Big\langle \exp\left[-2\pi i \big(\boldsymbol{\phi}, \boldsymbol{f}\big)\right] \Big\rangle_{\boldsymbol{f}}. \tag{1}$$

By explicit functional form, we mean an expression which does not involve the expectation angle brackets in (1). As mentioned in S1, one can modify (1) to admit spatiotemporal test functions $\boldsymbol{\phi} \equiv \phi(\boldsymbol{r}, t)$ by simply modifying the definition of the scalar product $\big(\boldsymbol{\phi}, \boldsymbol{f}\big)$ to incorporate time.

Gaussian Processes

The class of Gaussian processes is the most commonly known. Its complete statistical characterization relies on only the mean function $\bar{\boldsymbol{f}} = \bar{f}(\boldsymbol{r}, t)$ and the covariance function $\boldsymbol{k} = k(\boldsymbol{r}_1, \boldsymbol{r}_2, t)$ (see S1). First, note that the covariance function defines a *covariance operator* $\boldsymbol{\mathcal{K}}_t$, which acts on a test function $\boldsymbol{\phi}$ via

$$(\boldsymbol{\mathcal{K}}_t \boldsymbol{\phi})(\boldsymbol{r}_1) = \int_V d^3 r_2 \; k(\boldsymbol{r}_1, \boldsymbol{r}_2, t)\phi(\boldsymbol{r}_2)$$

Given $\bar{\boldsymbol{f}}$ and a valid $\boldsymbol{k}$, $\boldsymbol{f} \sim \mathcal{GP}(\bar{\boldsymbol{f}}, \boldsymbol{k})$ has characteristic functional [2, 3]

$$\Psi_{\boldsymbol{f}}[\phi(\boldsymbol{r}), t] = \exp\left[-2\pi i \big(\boldsymbol{\phi}, \bar{\boldsymbol{f}}\big)\right] \exp\left[-2\pi^2 \big(\boldsymbol{\mathcal{K}}_t \boldsymbol{\phi}, \boldsymbol{\phi}\big)\right] \tag{2}$$

where

$$\big(\boldsymbol{\phi}, \bar{\boldsymbol{f}}\big) = \int_V d^3 r \; \phi(\boldsymbol{r}) \bar{f}(\boldsymbol{r}, t), \;\; \big(\boldsymbol{\mathcal{K}}_t \boldsymbol{\phi}, \boldsymbol{\phi}\big) = \int_V d^3 r_1 \int_V d^3 r_2 \; k(\boldsymbol{r}_1, \boldsymbol{r}_2, t)\phi(\boldsymbol{r}_2)\phi(\boldsymbol{r}_1)$$

One of the key features of a Gaussian process is that any finite dimensional sample is multivariate Gaussian. Indeed, let $\boldsymbol{f} \sim \mathcal{GP}(\bar{\boldsymbol{f}}, \boldsymbol{k})$, $n \geq 1$, $\boldsymbol{r}_1, \ldots, \boldsymbol{r}_n \in V$, and $t \geq 0$. Then the random vector $\boldsymbol{X} = [f(\boldsymbol{r}_1, t), \ldots, f(\boldsymbol{r}_n, t)]$ has characteristic function

$$\begin{aligned}
\psi_{\boldsymbol{X}}(\boldsymbol{\xi}) =& \Psi_{\boldsymbol{f}} \left[\sum_{j=1}^{n} \xi_j \delta(\boldsymbol{r} - \boldsymbol{r}_j), t \right] \\
=& \exp\Big[- 2\pi i \sum_{j=1}^{n} \xi_j (\delta(\boldsymbol{r} - \boldsymbol{r}_j), \bar{\boldsymbol{f}}) \Big] \\
& \times \exp\left[-2\pi^2 \sum_{j,k=1}^{n} \xi_j \xi_k \left(\boldsymbol{\mathcal{K}_t} \delta(\boldsymbol{r} - \boldsymbol{r}_j), \delta(\boldsymbol{r} - \boldsymbol{r}_k) \right) \right] \\
=& \exp\left[-2\pi i \boldsymbol{\xi}^\intercal \overline{\boldsymbol{X}}\right] \exp\left[-2\pi^2 \boldsymbol{\xi}^\intercal \boldsymbol{C} \boldsymbol{\xi}\right]
\end{aligned} \tag{3}$$

where $^\intercal$ indicates the transpose and

$$\overline{\boldsymbol{X}}(i) = \bar{f}(\boldsymbol{r}_i, t), \quad C(i,j) = k(\boldsymbol{r}_i, \boldsymbol{r}_j, t). \tag{4}$$

Applying the inverse Fourier transform to (3), we obtain the PDF of $\boldsymbol{X}_{\boldsymbol{r}_1:\boldsymbol{r}_n,t}$:

$$p_{\boldsymbol{X}}(\mathbf{x}) = \frac{(2\pi)^{-n/2}}{\sqrt{\det(\boldsymbol{C})}} \exp\left(-\frac{1}{2}(\mathbf{x} - \overline{\boldsymbol{X}})^\intercal \boldsymbol{C}^{-1}(\mathbf{x} - \overline{\boldsymbol{X}})\right) \tag{5}$$

The finite dimensional distributions (5) provide a simple method to draw samples from $\mathcal{GP}(\bar{\boldsymbol{f}}, \boldsymbol{k})$: simply choose t and sample locations $\boldsymbol{r}_1, \ldots, \boldsymbol{r}_n \in V$, form $\overline{\boldsymbol{X}}$ and $\boldsymbol{C}$ using (4), then use an existing multivariate Gaussian sampler (based on e.g. Cholesky factorization) to draw from $\mathcal{N}(\overline{\boldsymbol{X}}, \boldsymbol{C})$. However, this can be very inefficient if n is large, for instance sampling a fine grid of points in 3D: Cholesky factorization is $O(n^3)$ and matrix multiplication is $O(n^2)$, so to draw K samples on a grid with m points per dimension, this method would be $O(m^9)$ for the first sample, then $O(Km^6)$ thereafter. Faster specialized methods are available if for instance $\boldsymbol{k}$ is shift-invariant; see e.g. [1].

In addition to the sample-based finite dimensional distributions (5), it is also useful to have the density of scalar random variables of the sort $X_{\boldsymbol{\phi},t} = (\boldsymbol{\phi}, \boldsymbol{f}) = \int_V d^3r\ \phi(\boldsymbol{r}) f(\boldsymbol{r}, t)$, i.e. a scalar product with a fixed test function. Such a random variable has (univariate) PDF $p_{\boldsymbol{\phi},t}$ given by

$$p_{\boldsymbol{\phi},t}(x) = \frac{1}{\sqrt{2\pi\sigma^2_{\boldsymbol{\phi},t}}} \exp\left(-\frac{(x - m_{\boldsymbol{\phi},t})^2}{2\sigma^2_{\boldsymbol{\phi},t}}\right) \tag{6}$$

where $\sigma_{\boldsymbol{\phi},t} = (\boldsymbol{\mathcal{K}}_t\boldsymbol{\phi}, \boldsymbol{\phi})$ and $m_{\boldsymbol{\phi},t} = (\boldsymbol{\phi}, \bar{\boldsymbol{f}})$, with $\boldsymbol{\mathcal{K}}$ and $\bar{\boldsymbol{f}}$ respectively the covariance operator and mean function of the process $\boldsymbol{f}$. Note that as the mean and covariance functions may depend on time, the PDF (6) also evolves in time.

One application of (2) is to provide a rigorous description of the *Gaussian white noise* process $\boldsymbol{w}$. While somewhat challenging to describe using standard probabilistic definitions, the characteristic functional provides a direct method to define a process which has mean zero and 'independent standard normal values at every point'. Intuitively, one would like the covariance function to be zero for $\boldsymbol{r}_1 \neq \boldsymbol{r}_2$, but nonzero for $\boldsymbol{r}_1 = \boldsymbol{r}_2$; the logical choice is a Dirac delta covariance, that is, $k(\boldsymbol{r}_1, \boldsymbol{r}_2, t) = \delta(\boldsymbol{r}_1 - \boldsymbol{r}_2)$. This leads to a covariance operator $\boldsymbol{\mathcal{K}}$ which is the identity operator, and hence the characteristic functional [4]

$$\Psi_{\boldsymbol{w}}[\boldsymbol{\phi}] = \exp(-2\pi^2(\boldsymbol{\phi}, \boldsymbol{\phi})) = \exp\left(-2\pi^2 \int_V d^3r\ |\phi(\boldsymbol{r})|^2\right)$$

A fairly wide variety of processes can be obtained under the Gaussian model by choosing different mean and covariance functions. The only requirement on $\bar{\boldsymbol{f}}$ and $\boldsymbol{k}$ is that $\boldsymbol{k}$ be *non-negative-type*, which means that any matrix of the form $C(i,j) = k(\boldsymbol{r}_i, \boldsymbol{r}_j, t)$ must be symmetric and non-negative definite i.e. a valid covariance matrix. Several common examples of covariance functions are provided in Table 1. In Figure 1 we display realizations for several choices of $\boldsymbol{k}$.

#	Name	Covariance function $\boldsymbol{k}$
(1)	Gaussian	$k(\boldsymbol{r}_1, \boldsymbol{r}_2; \sigma, \boldsymbol{A}) = \sigma^2 \exp\left(-\frac{1}{2}(\boldsymbol{r}_1 - \boldsymbol{r}_2)^t \boldsymbol{A}(\boldsymbol{r}_1 - \boldsymbol{r}_2)\right)$
(2)	Exponential	$k(\boldsymbol{r}_1, \boldsymbol{r}_2; \sigma, \ell) = \sigma^2 \exp(-\|\boldsymbol{r}_1 - \boldsymbol{r}_2\|/\ell)$
(3)	Matérn-Whittle	$k(\boldsymbol{r}_1, \boldsymbol{r}_2; \sigma, \nu, \ell) = \frac{\sigma^2}{2^{\nu-1}\Gamma(\nu)} \left(\frac{\|\boldsymbol{r}_1 - \boldsymbol{r}_2\|}{\ell}\right)^\nu K_\nu \left(\frac{\|\boldsymbol{r}_1 - \boldsymbol{r}_2\|}{\ell}\right)$
(4)	Bessel	$k(\boldsymbol{r}_1, \boldsymbol{r}_2; \sigma, \nu) = \sigma^2 \Gamma(d/2) \frac{J_\nu(\|\boldsymbol{r}_1 - \boldsymbol{r}_2\|)}{(\|\boldsymbol{r}_1 - \boldsymbol{r}_2\|/2)^\nu}$
(5)	White noise	$k(\boldsymbol{r}_1, \boldsymbol{r}_2) = \delta(\boldsymbol{r}_1 - \boldsymbol{r}_2)$

Table 1: A short list of common covariance functions used in spatial random process modeling, found in refs. [1, 5, 4, 6, 7]. The special functions $\Gamma(\cdot), K_\nu, J_\nu$ are respectively the Gamma function, modified Bessel function of the second kind, and Bessel function of the first kind. The matrix A in (1) must be symmetric nonnegative definite. Covariances (2)-(4) are their isotropic versions; anisotropic variants can also be defined. The covariance (5) must be interpreted as the generalized kernel of an integral operator.

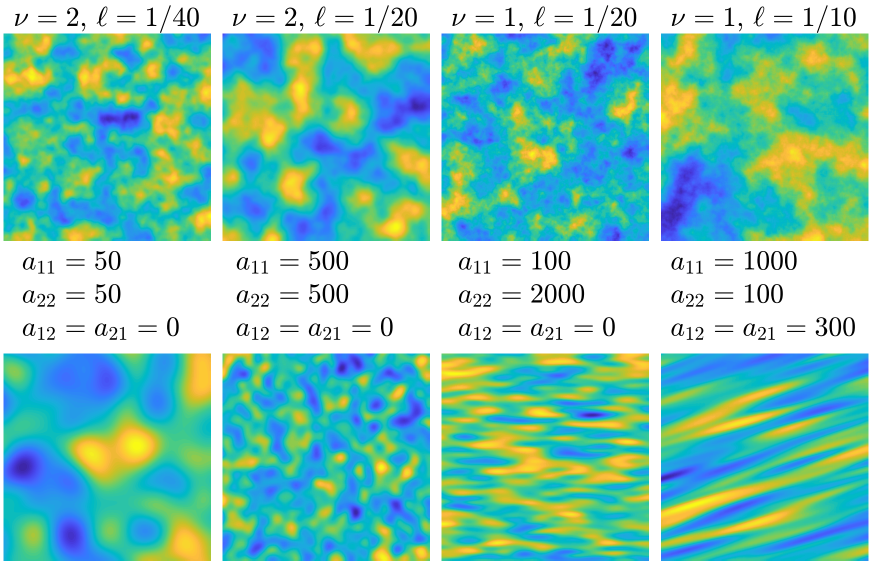

Figure 1: Simulated Gaussian random processes $f(\boldsymbol{r}, t)$ on $V = [0, 1]^2$. Top row uses the isotropic Matérn-Whittle covariance function (function (3) in Table 1) with parameters indicated above, while the bottom row uses the Gaussian covariance (function (1) in Table 1) with $A = (a_{ij})$ indicated above. All samples use variance $\sigma^2 = 1$ and were computed using Algorithm 7.6 in [1].

Lognormal Processes

One undesirable feature of the Gaussian model for physiology applications is that realizations are not guaranteed to be non-negative, whereas many physiological processes necessarily are. Given $\boldsymbol{z} \sim \mathcal{GP}(\bar{\boldsymbol{f}}, \boldsymbol{k})$, one possibility to easily generate other random processes from $\boldsymbol{z}$ is to consider point transformations of $\boldsymbol{z}$, i.e. we generate a process $\boldsymbol{f}$ by simply applying a deterministic function Υ to the realizations of $\boldsymbol{z}$:

$$f(\boldsymbol{r}, t, \omega) = \Upsilon(z(\boldsymbol{r}, t, \omega))$$

For the particular choice of $\Upsilon(z) = \exp(z)$, we form the class of *lognormal* processes, denoted $\mathcal{LN}(\bar{\boldsymbol{f}}, \boldsymbol{k})$, via

$$f(\boldsymbol{r}, t, \omega) = \exp(z(\boldsymbol{r}, t, \omega)), \quad \boldsymbol{z} \sim \mathcal{GP}(\bar{\boldsymbol{f}}, \boldsymbol{k})$$

It is well-known that the characteristic function of a lognormal random variable does not admit a closed-form representation [8], so similarly we cannot expect that a lognormal random process will admit a closed-form representation for the characteristic functional. In this work (namely S3), we were able to successfully manipulate $\boldsymbol{z} = \log \boldsymbol{f}$ using (2), so this was a non-issue.

Poisson Point Processes

A very useful class of non-Gaussian random process models are the various *point processes*. Realizations of a point process take the form

$$f(s) = \sum_{j=1}^{N} \delta(s - S_j) \tag{7}$$

where $S_1, \ldots, S_n$ is some collection of random states or sample points and N is a possibly random number of samples. We will consider only spatiotemporal *Poisson* point processes where $S_j = (\boldsymbol{R}_j, T_j)$ denotes a random spatiotemporal vector and the number of points N is a Poisson random variable. More general point processes are also possible [9, 3, 10].

To define a spatiotemporal Poisson Point Process (PPP), we require only an *intensity function* $\lambda(\boldsymbol{r}, t)$, which is a non-negative, integrable function on $V \times [0, \infty)$. We then define the mean number of points as

$$\overline{N} = \int_0^\infty \int_V \lambda(\boldsymbol{r}, t)\ d^3r dt < \infty$$

To form a point process of the sort (7), we first sample $N \sim \text{Poi}(\overline{N})$, where $\text{Poi}(\overline{N})$ is the Poisson distribution with rate $\overline{N}$. Then, we consider random variables $(\boldsymbol{R}_1, T_1), \ldots, (\boldsymbol{R}_N, T_N)$, independent and identically distributed with probability density

$$p(\boldsymbol{r}, t) = \frac{\lambda(\boldsymbol{r}, t)}{\overline{N}}$$

Given a realization $N = n$ and samples $(\boldsymbol{R}_1, T_1) = (\boldsymbol{r}_1, t_1), \ldots, (\boldsymbol{R}_n, T_n) = (\boldsymbol{r}_n, t_n)$, we form the point process as in (7):

$$f(\boldsymbol{r}, t) = \sum_{j=1}^{n} \delta(\boldsymbol{r} - \boldsymbol{r}_j)\delta(t - t_j) \tag{8}$$

Note that as (8) is a generalized function, one must take care in defining its statistical properties. For instance, the finite dimensional point sample vectors $\boldsymbol{X} = [f(\boldsymbol{r}_1, t), \ldots, f(\boldsymbol{r}_n, t)]$ become meaningless. However, the finite dimensional vectors $\boldsymbol{X} = [(\boldsymbol{\phi}_1, \boldsymbol{f}), \ldots, (\boldsymbol{\phi}_n, \boldsymbol{f})]$ and thus the characteristic functional is still well-defined, because of their definition in terms of test functions. To derive $\Psi_{\boldsymbol{f}}[\boldsymbol{\phi}]$ (which is now a spatiotemporal characteristic functional; see S1), we let $\boldsymbol{\phi} \equiv \phi(\boldsymbol{r}, t)$ be a (Schwartz class, say) test function. Then,

$$\big(\boldsymbol{\phi}, \boldsymbol{f}\big) = \sum_{j=1}^{N} \phi(\boldsymbol{R}_j, T_j)$$

where $N, \{\boldsymbol{R}_j\}$ and $\{T_j\}$ are the random quantities discussed above. Thus

$$\exp\left[-2\pi i\big(\boldsymbol{\phi}, \boldsymbol{f}\big)\right] = \prod_{j=1}^{N} \exp\left[-2\pi i\phi(\boldsymbol{R}_j, T_j)\right]$$

We then perform the expected value in (1) using the law of total probability and the definition of the random vector $(\boldsymbol{R}_j, T_j)$:

$$\begin{aligned}
\Psi_{\boldsymbol{f}}[\boldsymbol{\phi}] &= \left\langle \left\langle \exp\left[-2\pi i\big(\boldsymbol{\phi}, \boldsymbol{f}\big)\right] \right\rangle_{\{(\boldsymbol{R}_j, T_j)\}|N} \right\rangle_N \\
&= \left\langle \left(\int_0^\infty dt \int_V d^3r \;\exp(-2\pi i\phi(\boldsymbol{r}, t)) \frac{\lambda(\boldsymbol{r}, t)}{\overline{N}} \right)^N \right\rangle_N \\
&= \sum_{n=0}^{\infty} \frac{q_{\boldsymbol{\phi}}^n \exp(-\overline{N})\overline{N}^n}{\overline{N}^n n!} \\
&= \exp(-\overline{N}) \exp(q_{\boldsymbol{\phi}})
\end{aligned}$$

where we have defined $q_{\boldsymbol{\phi}} \equiv \int_0^\infty dt \int_V d^3r \;\exp(-2\pi i\phi(\boldsymbol{r}, t))\lambda(\boldsymbol{r}, t)$. Recalling the definition of $\overline{N}$, we can simplify once more to obtain

$$\Psi_{\boldsymbol{f}}[\boldsymbol{\phi}] = \exp\left[\int_0^\infty dt \int_V d^3r \;\left(\exp(-2\pi i\phi(\boldsymbol{r}, t)) - 1\right) \lambda(\boldsymbol{r}, t)\right]$$

We briefly discuss some extensions of the PPP model (8). First, we can consider a *filtered* version of (8), whereby we compute $g(\boldsymbol{r}, t) = (\mathcal{A}f)(\boldsymbol{r}, t)$ where $\mathcal{A}$ is a linear operator (defined for generalized functions). For instance, suppose

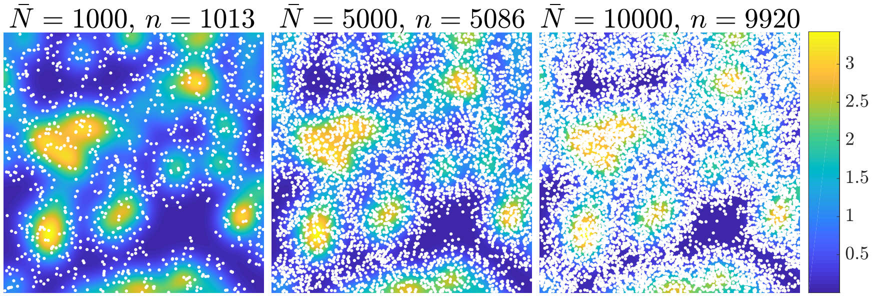

Figure 2: Simulation of three Poisson point processes with fixed PDF $p(\boldsymbol{r}, t) = \lambda(\boldsymbol{r}, t)/\bar{N}$ and varying $\bar{N}$. In each panel, a single realization of $f(\boldsymbol{r}, t)$ is displayed (white dots) over the color intensity plot of $p(\boldsymbol{r}, t)$. The mean number of points $\bar{N}$ and the realized number of points n are displayed above each panel. The PDF $p(\boldsymbol{r}, t)$ is a realization of a lumpy background process.

$h(\boldsymbol{r}, t)$ is a real, continuous, compactly supported function. Then, define $\boldsymbol{g} = \mathcal{A}\boldsymbol{f}$ to be the convolution of $\boldsymbol{f}$ with $h(\boldsymbol{r}, t)$:

$$\begin{aligned} g(\boldsymbol{r}, t) &= (h * f)(\boldsymbol{r}, t) \\ &= \int_0^\infty dt' \int_{\mathbb{R}^3} d^3r'\; h(\boldsymbol{r} - \boldsymbol{r}', t - t') \sum_{j=1}^{N} \delta(\boldsymbol{r}' - \boldsymbol{R}_j)\delta(t' - T_j) \\ &= \sum_{j=1}^{N} h(\boldsymbol{r} - \boldsymbol{R}_j, t - T_j) \end{aligned} \tag{9}$$

The characteristic functional of (9) then follows by the general relation for linearly transformed random processes (discussed in S1), namely that $\Psi_{\boldsymbol{g}}[\boldsymbol{\phi}] = \Psi_{\boldsymbol{f}}[\mathcal{A}^\dagger \boldsymbol{\phi}]$. With $\mathcal{A} = h*$, we have $\mathcal{A}^\dagger = \tilde{h}*$ where $\tilde{h}(\boldsymbol{r}, t) = h(-\boldsymbol{r}, -t)$, and so

$$\Psi_{\boldsymbol{g}}[\boldsymbol{\phi}] = \exp\left[\int_0^\infty dt \int_V d^3r\; \left(\exp(-2\pi i(\tilde{h} * \phi)(\boldsymbol{r}, t)) - 1\right) \lambda(\boldsymbol{r}, t)\right]$$

A particular example of (9) can be used to simulate extravascular drug diffusion component $\boldsymbol{c}^{diff}$ (see S4 and the main text). Let $h(\boldsymbol{r}, t)$ be the fundamental solution (Green's function) of a constant-coefficient diffusion equation, that is,

$$h(\boldsymbol{r}, t) = \frac{1}{(4\pi D_0 t)^{d/2}} \exp\left(-\frac{\|\boldsymbol{r}\|^2}{4D_0 t}\right)$$

then a solution to the diffusion equation with Poisson point process source $s(\boldsymbol{r}, t)$ results in a generalized Lumpy background process of the type (9):

$$c^{diff}(\boldsymbol{r}, t) = \sum_{j=1}^{n} h(\boldsymbol{r} - \boldsymbol{r}_j, t - t_j) = \sum_{j=1}^{N} \frac{1}{(4\pi D_0 (t - t_j))^{d/2}} \exp\left(-\frac{\|\boldsymbol{r} - \boldsymbol{r}_j\|^2}{4D_0 (t - t_j)}\right)$$

Taking (9) as a starting point, one can construct a wide array of possible random processes by choosing a different kernel functions $\boldsymbol{h}$, or even allowing multiple kernel functions $h_j(\boldsymbol{r}, t)$ in the sum (9). In general, we call a random process of the type

$$f(\boldsymbol{r}, t) = \sum_{j=1}^{N} h_j(\boldsymbol{r}, t; \boldsymbol{\theta}_j) \tag{10}$$

a *generalized lumpy background process.* The random variables that generate (10) are N and $(\boldsymbol{\theta}_1, \ldots, \boldsymbol{\theta}_N)$. The functions $h_j(\boldsymbol{r}, t; \boldsymbol{\theta})$ need not be the same type of function nor do they need to form a basis for any function space. Functions similar to (10) have been called *kernel density estimators* and *mixture models* [11], *shot noise* [10, 12], *lumpy backgrounds* [13, 14] and *texton noise* [15]. The model (10) can be evaluated in parallel at very high speed, and $\boldsymbol{h}_j$ can be chosen to match observed texture statistics [15, 16] or display desired regularity, nonnegativeness, boundedness, or any other desired sample function property. In particular, (10) can be made highly non-stationary and non-Gaussian, making it a particularly appealing class of models for physiological processes.

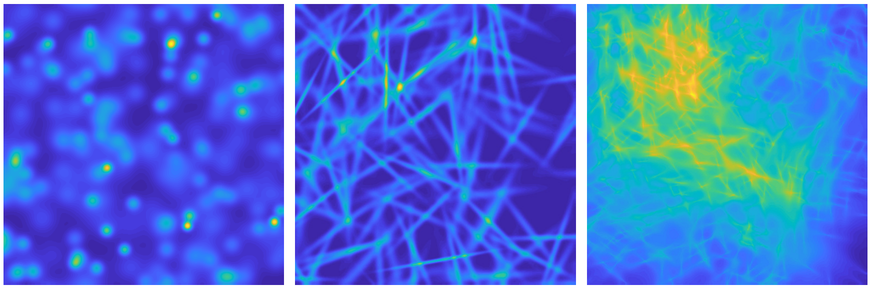

Figure 3: Three realizations of generalized lumpy background processes of the form (10) on the unit square $V = [0, 1]^2$. On the left, each lump takes the form $h_j(\boldsymbol{r}) = A_j \exp(-\|\boldsymbol{r}\|^2/2\sigma_j^2)$ with σ_j^2 drawn randomly from a lognormal distribution. In the middle, each lump takes the form $h_j(\boldsymbol{r}) = A \exp(-\boldsymbol{r}^t B_j \boldsymbol{r}/2)$ where B_j is a 2×2 positive definite matrix formed by rotating a matrix B_0 randomly. On the right is a *clustered* lumpy background [14] where each lump is of the form $h_j(\boldsymbol{r}) = \sum_{k=1}^{n_j} h(\boldsymbol{r} - \boldsymbol{r}_{jk})$.

The second direction we can generalize either (7), (9) or (10) is to allow the PPP intensity function $\boldsymbol{\lambda} \equiv \lambda(\boldsymbol{r}, t)$ to be a realization of a *secondary* random process, that is, we suppose that $\boldsymbol{\lambda}$ is a random process whose realizations are (almost surely) nonnegative and integrable. Then, if for each realization of $\boldsymbol{\lambda}$ we form a Poisson point process, we call the resulting random process a *doubly stochastic* or *Cox* Poisson point process [17, 9, 10], after which the filtered forms (9) or (10) can be formed.

References

[1] Lord GJ, Powell CE, Shardlow T. Introduction to Computational Stochastic PDEs. Cambridge; 2014.

[2] Clarkson E, Barrett HH. Characteristic functionals in imaging and image-quality assessment: tutorial. JOSA A. 2016;33(8):1464–1475.

[3] Barrett H, Myers K. Foundations of image science. John Wiley & Sons; 2004.

[4] Gel'fand IM, Vilenkin NY. Generalized functions, Vol. 4: applications of harmonic analysis. Academic Press; 1964.

[5] Christakos G. Random field models in earth sciences. Courier Corporation; 2012.

[6] Stein ML. Interpolation of spatial data: some theory for kriging. Springer Science & Business Media; 2012.

[7] Matérn B. Spatial variation. vol. 36. Springer Science & Business Media; 2013.

[8] Lukacs E. Characteristic functions. 1970;.

[9] Reiss RD. A course on point processes. Springer Science & Business Media; 2012.

[10] Snyder DL, Miller MI. Random point processes in time and space. Springer Science & Business Media; 2012.

[11] Silverman BW. Density estimation for statistics and data analysis. vol. 26. CRC press; 1986.

[12] Davenport WB, Root WL, et al. An introduction to the theory of random signals and noise. vol. 159. McGraw-Hill New York; 1958.

[13] Rolland JPY. Factors influencing lesion detection in medical imaging. The University of Arizona.; 1990.

[14] Bochud F, Abbey C, Eckstein M. Statistical texture synthesis of mammographic images with clustered lumpy backgrounds. Optics Express. 1999;4(1):33–42.

[15] Galerne B, Leclaire A, Moisan L. Texton noise. In: Computer Graphics Forum. Wiley Online Library; 2017.

[16] Kupinski MA, Clarkson E, Hoppin JW, Chen L, Barrett HH. Experimental determination of object statistics from noisy images. JOSA A. 2003;20(3):421–429.

[17] Cox DR. Some statistical methods connected with series of events. Journal of the Royal Statistical Society Series B (Methodological). 1955; p. 129–164.
