# Supplementary material for: Physiological random processes in precision cancer therapy
Source: PLoS One. 2018 Jun 29;13(6):e0199823. doi: 10.1371/journal.pone.0199823 (PMC6025881; doi:10.1371/journal.pone.0199823)
Supplement: S3 Appendix — (PDF) [file pone.0199823.s003.pdf]

S3 Appendix: Gompertzian tumor growth as a random process

For tumor growth in the absence of a chemotherapy drug, $c(\boldsymbol{r}, t) = 0$ and the equation for Gompetzian growth can be manipulated into the form

$$\frac{d}{dt} \ln n(\boldsymbol{r}, t) = \gamma(\boldsymbol{r}) - \mu(\boldsymbol{r}) \ln n(\boldsymbol{r}, t) \tag{1}$$

where $\gamma(\boldsymbol{r})$, defined as $\mu(\boldsymbol{r}) \ln n_{max}(\boldsymbol{r})$, is a time-independent random process. To maintain consistency with the Gompertzian model, we assume also that $\gamma(\boldsymbol{r})$ is statistically independent of $n(\boldsymbol{r}, t)$, since otherwise the statistics of the Gompertzian parameters $\mu(\boldsymbol{r})$ and $n_{max}(\boldsymbol{r})$ would have to depend on time. We also assume that the initial condition for (1) is given by a spatial random process $n_0(\boldsymbol{r}) \equiv n(\boldsymbol{r}, 0)$, independent from $\boldsymbol{\mu}$ and $\boldsymbol{\gamma}$.

For fixed $\boldsymbol{r}$, (1) is an ordinary first-order differential equation in t; the solution is

$$\ln n(\boldsymbol{r}, t) = \ln n_0(\boldsymbol{r}) \exp(-t\mu(\boldsymbol{r})) + \frac{\gamma(\boldsymbol{r})}{\mu(\boldsymbol{r})} \left[1 - \exp(-t\mu(\boldsymbol{r}))\right] , \tag{2}$$

or equivalently,

$$\ln n(\boldsymbol{r}, t) = \ln n_0(\boldsymbol{r}) \exp(-t\mu(\boldsymbol{r})) + \ln n_{max}(\boldsymbol{r}) \left[1 - \exp(-t\mu(\boldsymbol{r}))\right] . \tag{3}$$

To get a more familiar form for Gompertzian growth, we can exponentiate both sides of (3) and perform some algebra, yielding

$$n(\boldsymbol{r}, t) = n_0(\boldsymbol{r}) \exp\left[\ln\left(\frac{n_{max}(\boldsymbol{r})}{n_0(\boldsymbol{r})}\right) \left[1 - \exp(-t\mu(\boldsymbol{r}))\right]\right] . \tag{4}$$

For comparison, the usual Gompertzian growth formula is

$$N(t) = N_0 \exp\left[\ln\left(\frac{N_{max}}{N_0}\right) \left[1 - \exp(-t\mu)\right]\right] . \tag{5}$$

Note that we can derive (5) from (4) only by ignoring the dependences on spatial position $\boldsymbol{r}$, not by averaging over the volume of the tumor. We must implicitly assume that there is no spatial heterogeneity in the density of tumor cells or the growth parameters if we want to use just $N(t)$.

To describe this heterogeneity statistically, we need characteristic functionals. We have the option to compute the characteristic functional for either $n(\boldsymbol{r}, t)$ or $\ln n(\boldsymbol{r}, t)$; the latter is easier to compute and more relevant to tumor therapy.

In the vector-space notation introduced in S1, the characteristic functional for $\ln n(\boldsymbol{r}, t)$ is defined by

$$\Psi_{\ln \boldsymbol{n}}[\boldsymbol{\phi}, t] \equiv \Big\langle \exp\left[-2\pi i(\boldsymbol{\phi}, \ln \boldsymbol{n})\right] \Big\rangle_{\ln \boldsymbol{n}} , \tag{6}$$

where the time dependence is implicit in $\ln \boldsymbol{n}$ and $(\boldsymbol{\phi}, \ln \boldsymbol{n}) = \int_V d^3r\ \phi(\boldsymbol{r}) \ln n(\boldsymbol{r})$ is the scalar product of $\boldsymbol{\phi}$ and $\ln \boldsymbol{n}$.

We see from (3) that the random process $\ln \boldsymbol{n}$ depends on three other random processes: $\ln n_0(\boldsymbol{r})$, $\ln n_{max}(\boldsymbol{r})$ and $\mu(\boldsymbol{r})$, or in vector-space function notation, $\ln \boldsymbol{n} = F(\ln \boldsymbol{n}_0, \ln \boldsymbol{n}_{max}, \boldsymbol{\mu})$. Therefore, we can write

$$\begin{aligned}\Psi_{\ln \boldsymbol{n}}[\boldsymbol{\phi}, t] &\equiv \Big\langle \exp[-2\pi i(\boldsymbol{\phi}, \ln \boldsymbol{n})] \Big\rangle_{\ln \boldsymbol{n}_0, \ln \boldsymbol{n}_{max}, \boldsymbol{\mu}} \\ &= \Big\langle \Big\langle \Big\langle \exp\left[-2\pi i(\boldsymbol{\phi}, \ln \boldsymbol{n})\right] \Big\rangle_{\ln \boldsymbol{n}_0 |\ln \boldsymbol{n}_{max}, \boldsymbol{\mu}} \Big\rangle_{\ln \boldsymbol{n}_{max}|\boldsymbol{\mu}} \Big\rangle_{\boldsymbol{\mu}} . \qquad (7)\end{aligned}$$

With (3) and the assumption from above that $\ln \boldsymbol{n}_0$ and $\ln \boldsymbol{n}_{max}$ are statistically independent, we can write this characteristic functional as

$$\begin{aligned}\Psi_{\ln \boldsymbol{n}}[\boldsymbol{\phi}, t] = \Big\langle \Big\langle &\exp\Big[-2\pi i\Big(\boldsymbol{\phi}, \ln \boldsymbol{n}_0 \exp(-t\boldsymbol{\mu})\Big)\Big] \Big\rangle_{\ln \boldsymbol{n}_0} \\ &\times \Big\langle \exp\Big[-2\pi i\Big(\boldsymbol{\phi}, \ln \mathbf{n}_{max}[1 - \exp(-t\boldsymbol{\mu})]\Big)\Big] \Big\rangle_{\ln \boldsymbol{n}_{max}|\mu} \Big\rangle_{\mu} . \qquad (8)\end{aligned}$$

In this expression, $\ln \boldsymbol{n}_0 \exp(-t\boldsymbol{\mu})$ is to be interpreted as the function $\ln n_0(\boldsymbol{r}) \exp[-t\mu(\boldsymbol{r})]$. Thus the scalar product $\big(\boldsymbol{\phi}, \ln \boldsymbol{n}_0 \exp(-t\boldsymbol{\mu})\big)$ can be written as $\big(\boldsymbol{\phi} \exp(-t\boldsymbol{\mu}), \ln \boldsymbol{n}_0\big)$ simply by associating the exponential factor with $\phi(\boldsymbol{r})$ rather than $\ln n_0(\boldsymbol{r})$. With a similar manipulation on the second factor in (8), we see that

$$\begin{aligned}\Psi_{\ln \boldsymbol{n}}[\boldsymbol{\phi}, t] &= \Big\langle \Psi_{\ln \boldsymbol{n}|\mu}[\boldsymbol{\phi}, t] \Big\rangle_{\mu} \\ &= \Big\langle \Psi_{\ln \boldsymbol{n}_0}\left[\boldsymbol{\phi} \exp(-t\boldsymbol{\mu})\right] \cdot \Psi_{\ln \boldsymbol{n}_{max}|\mu}\left[\boldsymbol{\phi}\big(1 - \exp(-t\boldsymbol{\mu})\big)\right] \Big\rangle_{\mu} . \qquad (9)\end{aligned}$$

There is considerable evidence in the literature [1, 2] that the density of tumor cells is a lognormal random process, so we will now assume that the logarithm of the initial density, $\ln n_0(\boldsymbol{r})$, is a normal random process (see S2).

With the characteristic functional for a normal random process (see S2), we can write

$$\begin{aligned}\Psi_{\ln \boldsymbol{n}_0}\left[\boldsymbol{\phi} \exp(-t\boldsymbol{\mu})\right] = \exp[&-2\pi i(\boldsymbol{\phi}, \overline{\ln \boldsymbol{n}_0} \exp(-t\boldsymbol{\mu})] \\ &\times \exp[-2\pi^2(\boldsymbol{\phi}, \exp(-t\boldsymbol{\mu})\mathcal{K}_{\ln \boldsymbol{n}_0} \exp(-t\boldsymbol{\mu})\boldsymbol{\phi})] . \qquad (10)\end{aligned}$$

Because of the decaying exponential factors here, the characteristic functional of $\ln \boldsymbol{n}$ quickly loses its memory of the initial distribution $\ln \boldsymbol{n}_0$ as t increases. The remaining heterogeneity results from the randomness in $\ln \boldsymbol{n}_{max}$ and $\boldsymbol{\mu}$.

There is no direct information in the literature on the characteristic functionals of the Gompertzian parameters $\boldsymbol{n}_{max}$ and $\boldsymbol{\mu}$ because they have not previously been treated as random processes, but $\boldsymbol{n}_{max}$ should reflect tumor vascularity, which is often assumed to be lognormal. With that assumption, $\ln \boldsymbol{n}_{max}$ is a normal random process, and we see that [cf. (10)]

$$\Psi_{\ln \boldsymbol{n}_{max}|\mu}\left[\boldsymbol{\phi}\big(1 - \exp(-t\boldsymbol{\mu})\big)\right] \qquad (11)$$

$$\begin{aligned}&= \exp\Big[-2\pi i\big(\boldsymbol{\phi}, \overline{\ln \boldsymbol{n}_{max}}[1 - \exp(-t\boldsymbol{\mu})]\big)\big)\Big] \\ &\quad \times \exp\Big[-2\pi^2\Big(\boldsymbol{\phi}, [1 - \exp(-t\boldsymbol{\mu})]\mathcal{K}_{\ln \boldsymbol{n}_{max}}[1 - \exp(-t\boldsymbol{\mu})]\boldsymbol{\phi}\Big)\Big] . \qquad (12)\end{aligned}$$

It follows from the above that the conditional characteristic functional $\Psi_{\ln \boldsymbol{n}|\mu}(\boldsymbol{\phi}, t)$, defined by (9), is also normal, and the conditional mean vector and covariance operator are given by

$$\overline{\ln \boldsymbol{n}}(\mu) \equiv \langle \ln \boldsymbol{n} \rangle_{\ln \boldsymbol{n}_0, \ln \boldsymbol{n}_{max}|\mu} = \overline{\ln \boldsymbol{n}_0}\ \exp(-t\boldsymbol{\mu}) + \overline{\ln \boldsymbol{n}_{max}}\ [1 - \exp(-t\boldsymbol{\mu})] \quad (13)$$

$$\mathcal{K}_{\ln \boldsymbol{n}|\mu} = \exp(-t\boldsymbol{\mu})\mathcal{K}_{\ln \boldsymbol{n}_0} \exp(-t\boldsymbol{\mu}) + [1 - \exp(-t\boldsymbol{\mu})]\mathcal{K}_{\ln \boldsymbol{n}_{max}}[1 - \exp(-t\boldsymbol{\mu})] \quad (14)$$

It would be difficult to perform the remaining expectation over the growth rate $\boldsymbol{\mu}$ to go from $\Psi_{\ln \boldsymbol{n}|\mu}(\boldsymbol{\phi}, t)$ to $\Psi_{\ln \boldsymbol{n}}(\boldsymbol{\phi}, t)$ even if the characteristic functional for $\boldsymbol{\mu}$ were known, but there are two limits where that step is not necessary. As $t \to \infty$, $\exp(-t\boldsymbol{\mu}) \to 0$ and $[1 - \exp(-t\boldsymbol{\mu})] \to 1$; and if $t \to 0$, $\exp(-t\boldsymbol{\mu}) \to 1$ and $[1 - \exp(-t\boldsymbol{\mu})] \to 0$. In both cases $\boldsymbol{\mu}$ disappears, so $\Psi_{\ln \boldsymbol{n}}(\boldsymbol{\phi}, 0) = \Psi_{\ln \boldsymbol{n}_0}(\boldsymbol{\phi})$ and $\Psi_{\ln \boldsymbol{n}}(\boldsymbol{\phi}, \infty) = \Psi_{\ln \boldsymbol{n}_{max}}(\boldsymbol{\phi})$.

References

[1] Mackillop W, Stewart S, Buick R. Density/volume analysis in the study of cellular heterogeneity in human ovarian carcinoma. British journal of cancer. 1982;45(6):812–820.

[2] Akudugu JM, Neti PV, Howell RW. Changes in lognormal shape parameter guide design of patient-specific radiochemotherapy cocktails. Journal of Nuclear Medicine. 2011;52(4):642–649.
