# Supplementary material for: Physiological random processes in precision cancer therapy
Source: PLoS One. 2018 Jun 29;13(6):e0199823. doi: 10.1371/journal.pone.0199823 (PMC6025881; doi:10.1371/journal.pone.0199823)
Supplement: S4 Appendix — (PDF) [file pone.0199823.s004.pdf]

S4 Appendix: Characteristic functionals for diffusion

As noted in the main text, the general form of the diffusion equation is

$$\frac{\partial}{\partial t} c^{diff}(\boldsymbol{r},t) - \nabla \cdot \left[D(\boldsymbol{r}) \nabla c^{diff}(\boldsymbol{r},t) \right] = s(\boldsymbol{r},t)\,, \tag{1}$$

where $\equiv D(\boldsymbol{r})$ is the diffusion coefficient, treated as a time-independent spatial random process, and $s(\boldsymbol{r},t)$ is a random source term related to vascular permeability and drug concentration in the tumor capillaries.

Defining $\boldsymbol{D} \equiv D(\boldsymbol{r})$, $\boldsymbol{c}(t) \equiv c(\boldsymbol{r},t)$ and $\boldsymbol{s}(t) \equiv s(\boldsymbol{r},t)$, we can write the diffusion equation above in vector-space form as

$$\frac{\partial \boldsymbol{c}^{diff}(t)}{\partial t} + \mathcal{D}\boldsymbol{c}^{diff}(t) = \mathbf{s}(t)\,, \tag{2}$$

where

$$\mathcal{D} \equiv -\nabla \cdot \boldsymbol{D} \nabla\,. \tag{3}$$

Now multiply from the left by the exponential operator $\exp(t\mathcal{D})$; the result is

$$\frac{\partial}{\partial t}\left[\exp(t\mathcal{D})\boldsymbol{c}^{diff}(t)\right] = \exp(t\mathcal{D})\mathbf{s}(t)\,. \tag{4}$$

Integrate both sides 0 to t, assuming $\boldsymbol{c}^{diff}(0) \equiv 0$:

$$\exp(t\mathcal{D})\boldsymbol{c}^{diff}(t) = \int_0^t dt' \exp(t'\mathcal{D})\mathbf{s}(t')\,. \tag{5}$$

When we multiply from the left by $\exp(-t\mathcal{D})$, which is the inverse of the operator $\exp(t\mathcal{D})$, we obtain

$$\boldsymbol{c}^{diff}(t) = \int_0^t dt' \exp[(t'-t)\mathcal{D}]\mathbf{s}(t')\,. \tag{6}$$

With this result we can write the characteristic functional for $\boldsymbol{c}^{diff}$ as

$$\begin{aligned} \boldsymbol{\Psi}_{\boldsymbol{c}^{diff}}(\boldsymbol{\phi},t) &= \left\langle \left\langle \left\langle \exp\left[-2\pi i \left(\boldsymbol{\phi}, \boldsymbol{c}^{diff}(t)\right)\right] \right\rangle_{\boldsymbol{c}^{diff}|\mathbf{s},\boldsymbol{D}} \right\rangle_{\mathbf{s}|\boldsymbol{D}} \right\rangle_{\boldsymbol{D}} \\ &= \left\langle \left\langle \exp\left[-2\pi i \int_V d^3r\, \phi(\boldsymbol{r}) \int_0^t dt' \exp[(t'-t)\mathcal{D}]\mathbf{s}(t')\right] \right\rangle_{\mathbf{s}} \right\rangle_{\boldsymbol{D}} \end{aligned} \tag{7}$$

In the second line we have assumed that $\mathbf{s}$ and $\boldsymbol{D}$ are statistically independent; there is no reason for vascular flow and permeability to depend on diffusion outside the vessels.

Because the adjoint of an exponential operator is the exponential of the adjoint, we can also write

$$\left[\exp\left(t\mathcal{D}\right)\right]^{\dagger} = \exp\left(t\mathcal{D}^{\dagger}\right)\,. \tag{8}$$

Moreover, we can think of the integral over t' in (7) as a Riemann sum and use the fact that the adjoint of a sum is the sum of the adjoints, so

$$\left[\int_0^t dt' \exp[(t'-t)\mathcal{D}]\right]^\dagger = \int_0^t dt' \exp[(t'-t)\mathcal{D}^\dagger]\,. \tag{9}$$

With these manipulations, (7) becomes

$$\begin{aligned}\mathbf{\Psi}_{\boldsymbol{c}^{diff}}(\boldsymbol{\phi},t) &= \left\langle\left\langle \exp\left[-2\pi i\int_V d^3r\,\phi(\boldsymbol{r})\int_0^t dt' \exp[(t'-t)\mathcal{D}](s(\boldsymbol{r},t')\right]\right\rangle_{\mathbf{s}}\right\rangle_{\boldsymbol{D}} \\ &= \left\langle\left\langle \exp\left[-2\pi i\int_V d^3r\,s(\boldsymbol{r},t)\int_0^t dt' \exp[(t'-t)\mathcal{D}^\dagger]\phi(\boldsymbol{r})\right]\right\rangle_{\mathbf{s}}\right\rangle_{\boldsymbol{D}} \end{aligned}\tag{10}$$

or equivalently

$$\mathbf{\Psi}_{\boldsymbol{c}^{diff}}(\boldsymbol{\phi},t) = \left\langle \mathbf{\Psi}_{\mathbf{s}}\Big(\int_0^t dt' \exp[(t'-t)\mathcal{D}^\dagger]\boldsymbol{\phi}\Big)\right\rangle_{\boldsymbol{D}}. \tag{11}$$

Thus we can find the characteristic functional for the diffusing component of the drug concentration if we have the characteristic functional of the random source as well as a way of performing the final average over $\boldsymbol{D}$.

To perform calculations with (11), we need an explicit form for $\mathcal{D}^\dagger$. With the help of standard identities from 3D vector analysis, $\mathcal{D}$ itself can can be written as

$$\mathcal{D}s(\boldsymbol{r}) = -\nabla\cdot[D(\boldsymbol{r})\nabla s(\boldsymbol{r})] = -D(\boldsymbol{r})\nabla^2 s(\boldsymbol{r}) - \nabla D(\boldsymbol{r})\cdot\nabla s(\boldsymbol{r})\,. \tag{12}$$

The adjoint of this operator is given by

$$\mathcal{D}^\dagger\phi(\boldsymbol{r}) = -\nabla^2[D(\boldsymbol{r})\phi(\boldsymbol{r})] + \nabla\cdot[\phi(\boldsymbol{r})\nabla D(\boldsymbol{r})]\,. \tag{13}$$

By expressing the divergence and gradient in Cartesian coordinates and performing an integration by parts, it can be verified that

$$\int_V d^3r\,\phi(\boldsymbol{r})\mathcal{D}s(\boldsymbol{r}) = \int_V d^3r\,s(\boldsymbol{r})\mathcal{D}^\dagger\phi(\boldsymbol{r})\,. \tag{14}$$

In the special case where $\boldsymbol{D}\equiv D_0$ is a constant so that $\nabla D(\boldsymbol{r}) = 0$ and $\mathcal{D} = -D_0\nabla^2$, the linear operator $\mathcal{D}$ is Hermitian and shift-invariant and the scalar products we need are easily computed in the 3D Fourier domain, where the diffusion operator acts as a low-pass filter:

$$\begin{aligned}&\int_0^t dt' \exp[(t'-t)\mathcal{D}^\dagger]\boldsymbol{\phi}(\boldsymbol{r}) \\ &\qquad= \int_0^t dt'\,\exp\left[(t-t')D_0\nabla^2\right]\int_\infty d^3\rho\,\Phi(\boldsymbol{\rho})\,\exp(2\pi i\boldsymbol{\rho}\cdot\boldsymbol{r}) \\ &\qquad= \int_\infty d^3\rho\,\Phi(\boldsymbol{\rho})\int_0^t dt'\,\exp\left[-4\pi^2(t-t')D_0\rho^2\right]\exp(2\pi i\boldsymbol{\rho}\cdot\boldsymbol{r})\,. \end{aligned}\tag{15}$$

Note that $t-t'$ is nonnegative, so $\exp\left[-4\pi^2(t-t')D_0\rho^2\right]$ is a 3D Gaussian low-pass filter with a time-dependent width.
